# A systematic review of cost-effectiveness studies comparing conventional, biological and surgical interventions for inflammatory bowel disease

**DOI:** 10.1371/journal.pone.0185500

**Published:** 2017-10-03

**Authors:** Nadia Pillai, Mark Dusheiko, Bernard Burnand, Valérie Pittet

**Affiliations:** 1 Institute of Social and Preventive Medicine (IUMSP), Lausanne University Hospital, Lausanne, Switzerland; 2 Centre for Health Economics, University of York, York, United Kingdom; 3 Faculty of Business and Economics (HEC), University of Lausanne, Lausanne, Switzerland; 4 Cochrane Switzerland, Institute of Social and Preventive Medicine (IUMSP), Lausanne University Hospital, Lausanne, Switzerland; University Hospital Llandough, UNITED KINGDOM

## Abstract

**Background:**

Inflammatory bowel disease (IBD) is a chronic disease placing a large health and economic burden on health systems worldwide. The treatment landscape is complex with multiple strategies to induce and maintain remission while avoiding long-term complications. The extent to which rising treatment costs, due to expensive biologic agents, are offset by improved outcomes and fewer hospitalisations and surgeries needs to be evaluated. This systematic review aimed to assess the cost-effectiveness of treatment strategies for IBD.

**Materials and methods:**

A systematic literature search was performed in March 2017 to identify economic evaluations of pharmacological and surgical interventions, for adults diagnosed with Crohn’s disease (CD) or ulcerative colitis (UC). Costs and incremental cost-effectiveness ratios (ICERs) were adjusted to reflect 2015 purchasing power parity (PPP). Risk of bias assessments and a narrative synthesis of individual study findings are presented.

**Results:**

Forty-nine articles were included; 24 on CD and 25 on UC. Infliximab and adalimumab induction and maintenance treatments were cost-effective compared to standard care in patients with moderate or severe CD; however, in patients with conventional-drug refractory CD, fistulising CD and for maintenance of surgically-induced remission ICERs were above acceptable cost-effectiveness thresholds. In mild UC, induction of remission using high dose mesalazine was dominant compared to standard dose. In UC refractory to conventional treatments, infliximab and adalimumab induction and maintenance treatment were not cost-effective compared to standard care; however, ICERs for treatment with vedolizumab and surgery were favourable.

**Conclusions:**

We found that, in general, while biologic agents helped improve outcomes, they incurred high costs and therefore were not cost-effective, particularly for use as maintenance therapy. The cost-effectiveness of biologic agents may improve as market prices fall and with the introduction of biosimilars. Future research should identify optimal treatment strategies reflecting routine clinical practice, incorporate indirect costs and evaluate lifetime costs and benefits.

## Introduction

Inflammatory bowel disease (IBD) refers mainly to Crohn’s disease (CD) and ulcerative colitis (UC), which are chronic, autoimmune conditions causing inflammation in the gastrointestinal tract and extra-intestinal complications. IBD follows a course of exacerbation and remission of inflammation with symptoms characterised by chronic abdominal pain, diarrhoea and weight loss [1].

The clinical management of IBD aims to induce and maintain remission in patients with active disease [2]. Treatment strategies are complex, consisting of pharmacological treatment and surgery depending on disease location, severity and patients’ treatment history [3]. The traditional step-up approach consists of first-line therapy with “conventional” or standard of care treatments such as aminosalicylates, corticosteroids, and immunomodulators (e.g. azathiopurine, 6-mercaptopurine) [4]. More recently, biologic agents are being used to induce remission in patients with moderate to severe disease and disease which responds poorly or is refractory to conventional medicines [5, 6]. Anti-tumour necrosis factor (TNF) agents, infliximab, adalimumab, and golimumab are approved for use in CD and UC by the European Medicine’s Agency (EMA) and the USA Food and Drug Administration (FDA); certolizumab pegol is approved only for CD in Switzerland, the USA and Russia [7]. In addition, two anti-integrin molecules are available: vedolizumab, approved in the USA and Europe for CD and UC, and natalizumab, approved in the USA for CD only. These agents provide promising alternatives to conventional medications as they are associated with reduced dependence on corticosteroids as well as longer duration of remission and improved overall quality of life [8].

IBD is among the top five most expensive gastrointestinal disorders to treat; it incurs wider social costs and reduces patients’ quality of life [9]. Within Europe, estimates from 2013 suggest that 2.5–3 million people are affected with IBD contributing an overall direct health care cost of 4.6–5.6 billion Euros per year [10]. These figures are higher in the USA, which has an estimated prevalence of 214 per 100,000 individuals for CD and UC each [6, 11]. The increasing prevalence, high morbidity and costs of IBD represent an important challenge, requiring resources and infrastructure for efficient long-term chronic disease management [11, 12].

The economic burden of IBD is changing whereby costs are increasingly driven by biologic agents and less by hospitalisations and surgery [13]. Despite the high costs of biologic agents, increasing use of these agents is seen due to their efficacy [14]. Given the uncertainties around the optimal use of biologic agents in IBD, increased scrutiny on the cost-effectiveness of different treatment strategies is required to aid cost-containment discussions while still ensuring patients’ receive the best available treatments. Economic evaluations aim to compare alternative strategies by relating the improvement in health outcomes to the overall treatment costs across health states and over time in order to inform decision-making on the optimal use of available resources [15]. We conducted a systematic literature review of the cost-effectiveness of pharmacological or surgical interventions in adults diagnosed with CD or UC across different health systems and a spectrum of clinical presentations. The objective of this review was to provide an understanding of the cost-effective treatment strategies, particularly the biologic agents, and identify gaps in the literature and requirements for future economic models in IBD.

## Materials and methods

### Literature search

An extensive literature search was performed on 16 November 2016 and updated on 21 March 2017 in key databases: Ovid Medline (1946 to present), Embase (1974 to Nov 14, 2011), Database of Abstracts of Reviews of Effects (DARE, 1994 to March 2015), National Health Service Economic Evaluation Database (NHS EED, 1994 to March 2015), and Health Technology Assessment (HTA). Search terms used were: Crohn’s disease, ulcerative colitis, inflammatory bowel disease, cost effectiveness, cost utility, cost benefit, health economic, economic evaluation (see S1 Table for detailed search strategy). Searches were limited to articles published in English and no date limits were applied. Attempts were made to identify full texts for any conference abstracts, however, where none were available, the abstracts were excluded due to insufficient information reported. In addition, a manual search of references from identified literature was performed. All references were downloaded to EndNote X8 and duplicates were removed.

### Study selection

Title, abstract and full-text screening was conducted by NP. Studies were included in the review according to the PICOS (population, intervention, comparator, outcomes and study design) criteria. Full economic evaluations (cost-effectiveness, cost-benefit and cost-utility analyses) were included in the review if they included adults (aged ≥18), diagnosed with CD or UC, and compared surgical or pharmacological interventions. Models from drug manufacturers reported in HTA submissions were also included provided sufficient detail was available. Studies were excluded from the review if they were partial economic evaluations, if they did not specifically evaluate treatments for IBD or if they were a letter, comment piece or editorial.

### Data extraction and interpretation

Data extraction was conducted based on guidance from the Cochrane Handbook [16]. Data extracted included disease indication, year and setting, intervention and comparator, perspective, study design, type of decision analysis (e.g. Markov model or decision tree analysis), time horizon, source and year of costs, currency, discount rate, source of outcomes and benefits, sensitivity analysis, and study results. To aid comparisons, costs were inflated to 2015 prices in US Dollars, using the OECD consumer price index (CPI) [17], and then converted to 2015 purchasing power parity (PPP) using OECD rates [18]. Where the year of cost data collection was not reported the year of publication was used instead.

The overall cost-effectiveness result, normally expressed in terms of an incremental cost-effectiveness ratio (ICER), represents the additional cost per unit of effectiveness (often the quality-adjusted life year (QALY)) achieved from adopting one intervention relative to an alternative. The ICER was recalculated to reflect 2015 PPP costs per unit of effectiveness, using the following formula:

$$ICER=\frac{PPP\;Cost\;of\;intervention\;1–PPP\;Cost\;of\;intervention\;2}{Effectiveness\;of\;intervention\;1–Effectiveness\;of\;intervention\;2}$$

When interpreting the ICER, interventions were said to be dominant (or dominated) if the costs of intervention 1 were lower (or higher) and its effectiveness better (or worse) than intervention 2. When both the costs and effectiveness of intervention 1 were higher (or lower) a threshold at which the cost of obtaining an additional unit of effectiveness (or savings for the loss of effectiveness) is acceptable was normally used. In the UK, the National Institute for Clinical Excellence (NICE) recommends a technology or drug as cost-effective if it has an ICER between 20’000 GBP to 30’000 GBP per QALY gained (29’069.77–43’604.65 in 2015 PPP), reflecting the opportunity cost incurred of obtaining an additional QALY had the money been spent elsewhere in the health system [19]. In the USA, a threshold of 100’000 USD to 150’000 USD has been informally accepted by decision-makers and researchers based on estimated values of an additional statistical life year [20]. These thresholds are still contested and subject to change [21–23], therefore, in this study, conclusions drawn with respect to cost-effectiveness reflect the setting of the original study.

### Risk of bias

As recommended by available guidelines, bias assessments were performed using the Drummond et al. (2006) checklist [24] for economic evaluations and the checklist from Philips et al. (2004) for model-based economic evaluations [25] [26].

### Study synthesis

This systematic review presents a narrative summary discussing studies on CD and UC by clinical presentation (mild, moderate, severe, disease refractory to conventional treatments, fistulising CD, and surgically-induced remission) and treatment aims (induction, maintenance and both induction and maintenance). A descriptive analysis of the studies is presented followed by the results of cost-effectiveness for individual studies. Based on recommendations from guidelines for systematic reviews in economic evaluations, no attempts were made to quantitatively pool study results [26].

## Results

### Study selection

**Fig 1:**
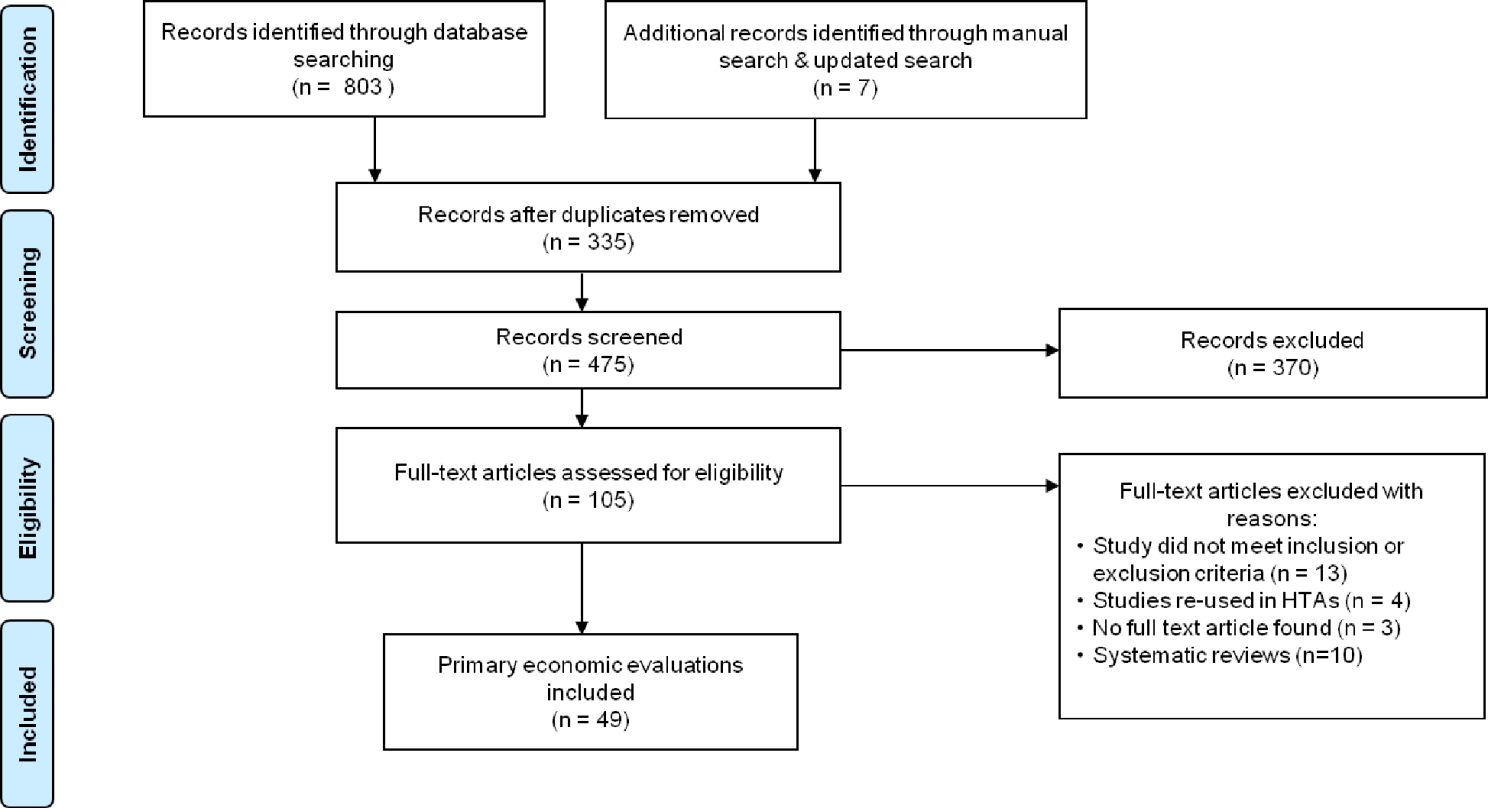
Flow chart of study inclusion based on the Preferred Reporting Items for Systematic Reviews and Meta-Analyses (PRISMA)[27].

The literature search revealed 803 records of which 49 full text articles were retained after removing duplicates and applying the inclusion and exclusion criteria (See Fig 1). Of the included studies, 24 focus on CD and 25 on UC.

### Descriptive analysis

**Fig 2:**
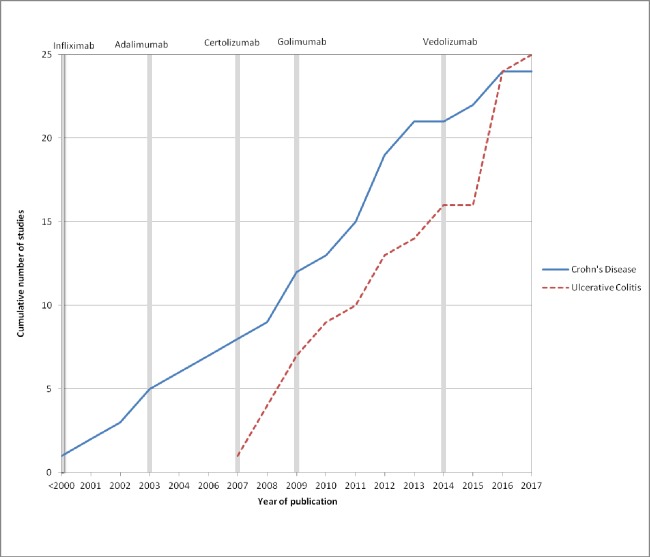
Frequency of published economic evaluations on Crohn’s disease and ulcerative colitis over time; grey bars indicate year of market approval by the European Medicines Agency (EMA).

An increasing number of economic evaluations in IBD have been published over the past 20 years (see Fig 2). The oldest study identified on CD was published in 1997, while, the majority were published from 2000, following the market approval of infliximab. A large increase in economic evaluations on UC was seen in 2016; however, the first publication identified was in 2007. This reflects both the increasing number of novel pharmacological agents for IBD as well as the uptake of economic evaluations in healthcare.

Heterogeneous methods were used to generate cost-effectiveness results across studies (Table 1). For example, a time horizon of one year or less was used in more than 50% of the studies on CD but in only 36% of studies on UC. Only 21% and 24% of studies on CD and UC, respectively, used the recommended lifetime time horizon. Secondly, studies mostly adopted the health system perspective, particularly the third party payer and the publically funded health system, reflecting the USA and UK health systems, where the majority of studies were conducted. Two studies on CD and three studies on UC reported adopting a societal perspective (i.e. incorporating indirect costs to the patient in the model); however, in three of these studies no evidence of indirect costs were found in the publications [28–30]. Studies also differed in the type of decision analysis used (e.g. static decision analytic models versus Markov models). Finally, most studies used QALYs as the main effectiveness measure, creating a cost-utility analysis; while, two studies on CD and two studies on UC undertook a cost-effectiveness analysis, using outcomes such as number of patients in remission [31, 32], number of surgeries [32], time spent in remission [33] and the probability of achieving mucosal healing (MH) [34].

**Table 1:** Key characteristics of published economic evaluations in Crohn’s disease and ulcerative colitis.

Characteristics	Crohn's Disease (N, %)	Ulcerative Colitis (N, %)
**Time horizon**		
Lifetime	5 (21%)	6 (24%)
10 years	1 (4%)	5 (20%)
5 years	3 (13%)	4 (16%)
2 years	1 (4%)	1 (4%)
1 year	13 (54%)	6 (24%)
32 weeks	0	1 (4%)
12 weeks	0	1 (4%)
Not stated	0	1 (4%)
Other	1 (4%)	0
**Setting**		
USA	10 (42%)	4 (16%)
UK	8 (33%)	11 (44%)
Canada	2 (8%)	2 (8%)
Other	4 (17%)	8 (32%)
**Study design**		
Cost-effectiveness analysis	2 (8%)	2 (8%)
Cost-utility analysis	22 (92%)	23 (92%)
**Type of decision analysis**		
Decision analytic model	8 (33%)	7 (28%)
Markov model	12 (50%)	16 (64%)
Monte Carlo simulation	2 (8%)	0
Markov cohort model	1 (4%)	2 (8%)
Cohort model not clearly defined	1 (4%)	0
**Perspective**		
Third party payer	11 (46%)	6 (24%)
Publically-funded health system	8 (33%)	16 (64%)
Societal	2 (8%)	3 (12%)
Not clear	3 (13%)	0

### Crohn’s disease

The results of the 24 studies on CD are summarised in Table 2.

**Table 2:** Summary of cost-effectiveness results adjusted to 2015 PPP for studies on Crohn’s disease.

Reference (year, country)	Clinical presentation	Interventions/Comparators*	Inflated cost (2015 PPP)	Outcome (QALY unless otherwise stated)	ICER (PPP per outcome gained)†
**Trallori et al. (1997, unclear) [30]**	Patients in remission	Maintenance therapy with mesalazine	8'578'448.72	1713.6	8'471.74
		No maintenance treatment	8'417'485.58	1694.6	Reference
**Arsenau et al. (2001, USA) [35]**	Initial treatment of perianal fistula	6MP /metronidazole combination	4'118.09	0.76	Reference
		Initial infliximab induction infusions plus combination with 6MP/metronidazole if treatment failure	14'234.03	0.78	505'796.84
		Initial infliximab induction infusions with episodic reinfusion if treatment failure	14'389.13	0.78	513'552.06
		6MP/metronidazole followed by infliximab induction infusions with episodic reinfusion if treatment failure	9'482.71	0.77	536'461.97
**Marshall et al. (2002, Canada) [36]**	Active disease refractory to conventional therapies	Strategy A: “usual care” immunosuppressants, intravenous corticosteroids and surgery	10'278.04	0.6281	Strategy A vs. Strategy B: 187'890.19
		Strategy B: Single infliximab infusion at week 0	13'133.97	0.6433	Strategy C vs. Strategy B: 487'393.91
		Strategy C: Single infliximab infusion at week 0 plus reinfusion for patients who relapse	14'206.24	0.6455	Strategy D vs. Strategy C: 719'047.53
		Strategy D: Single infliximab infusion at week 0 plus maintenance infliximab for patients who respond and usual care for patients who do not respond	22'331.48	0.6568	-
**Clark et al. (2003, UK) Schering-Plough model [37]**‡	Chronic active disease refractory to conventional therapies	Single infliximab infusion			9'738.37
		Episodic infliximab infusions *(timing not stated)*			15'116.28
		Maintenance infliximab infusions *(timing not stated)*			122'674.42
		Placebo			Reference
**Clark et al. (2003, UK) Schering-Plough model [37]**‡	Fistulising Crohn’s disease	Initial infliximab induction infusions			178'779.07
		Initial infliximab induction infusions plus retreatment if fistula reopens			139'534.88
		Initial infliximab induction infusions plus maintenance treatment for patients achieving 100% fistula closure			170'058.14
		Placebo			Reference
**Clark et al. (2003, UK) Primary economic evaluation [37]**‡	Chronic active disease refractory to conventional therapies	Infliximab 5mg/kg single infusion			135'529.07
		Infliximab 5mg/kg episodic infusions (three re-treatments)			90'139.53
		Infliximab (5, 10 and 20mg/kg doses) single infusion			196'704.94
		Infliximab (all doses) episodic (three re-treatments)			105'030.52
		Placebo			Reference
**Jaisson-Hot et al. (2004, France) [38]**§	Moderate to severe active ileocolonic disease refractory to conventional therapies	Strategy 1a: Initial infliximab infusion plus re-treatment when patients relapse or do not respond	173'478.98	30.78	60'550.01
		Strategy 1b: Initial infliximab infusion plus maintenance infliximab infusions every 8 weeks	994'937.83	30.78	768'704.19
		Strategy 2: Surgery	103'240.97	29.62	Reference
Priest et al. (2006, NZ) [39]**	Moderate to severe CD indicated for immuno-suppressive therapy	Azathioprine maintenance therapy	1'220'732.02	877.6	Azathioprine dominant
		Methotrexate maintenance therapy	1'493'388.54	633.4	Reference
**Kaplan et al. (2007, USA) [32]**	Moderate to severe disease after loss of response during maintenance infliximab treatment	Infliximab dose escalation to 10mg/kg every 8 weeks	33'349.18	0.79	403'359.61
		Discontinue infliximab and switch to adalimumab induction and maintenance therapy	21'248.39	0.76	
**Lindsay et al. (2008, UK) [40]**	Moderate to severe active luminal disease	Infliximab initial infusions and maintenance treatment	58'626.42	2.145	48'751.83
		Standard care (immunomodulators and/or corticosteroids)	49'558.58	1.959	
**Lindsay et al. (2008, UK) [40]**	Fistulising Crohn’s Disease	Infliximab initial infusions and maintenance therapy	69'773.24	2.449	55'265.19
		Standard care (immunomodulators and/or corticosteroids)	58'609.67	2.247	
**Bodger et al. (2009, UK) [41]**	Moderate to severe active disease	Infliximab infusions for induction of remission followed by maintenance treatment for 1 year	91'568.88	14.568	34'664.32
		Infliximab infusions for induction of remission followed by maintenance treatment for 2 years	105'941.90	14.901	38'753.63
		Adalimumab injection for induction of remission followed by maintenance treatment for 1 year	85'019.15	14.682	12'462.49
		Adalimumab injection for induction of remission followed by maintenance treatment for 2 year	96'590.34	15.156	18'443.45
		Standard care (5ASA, immunosuppressive agents, corticosteroids, antibiotics, symptomatic therapies, topical therapies and surgery)	79'124.39	14.209	Reference
**Loftus et al. (2009, UK) [42]**	Severe active disease	Adalimumab induction and maintenance therapy injection	19'798.38	0.8516	29'215.03
		Non-biologic therapy (based on the CLASSIC I trial: placebo and conventional medications)	16'359.77	0.7339	Reference
	Moderate to severe active disease	Adalimumab induction and maintenance therapy injection	17'640.61	0.8647	61'323.23
		Non-biologic therapy (based on the CLASSIC I trial: placebo and conventional medications)	12'096.99	0.7743	Reference
**Yu et al. (2009, USA) [43]**	Moderate to severe active disease	Adalimumab induction and maintenance injections	40'198.41	0.865	Adalimumab dominant
		Infliximab induction and maintenance infusions	45'902.58	0.851	
**Bakhshai et al. (2010, USA) [33]**§	Moderate to severe active disease eligible for second line biologic therapy	Natalizumab induction and maintenance infusion	74'316.05	4.5 months in remission	Reference
		Infliximab induction and maintenance infusions	67'487.91	2.4 months in remission	Dominated by adalimumab
		Adalimumab induction and maintenance injection	67'168.35	2.88 months in remission	4412.16 per month of remission
**Dretzke et al. (2011, UK) [44]**	Severe active disease refractory to conventional therapies	Standard care	24'406.85	0.8119	Dominated
		Infliximab induction infusions	21'925.23	0.8943	Reference
		Infliximab maintenance infusions	34'828.20	0.8957	9'216'407.48
	Severe active disease refractory to conventional therapies	Standard care	24'417.76	0.8118	Dominated
		Adalimumab induction infusions	12'832.02	0.8942	Reference
		Adalimumab maintenance infusions	25'556.69	0.8956	9'089'051.59
	Moderate active disease refractory to conventional therapies	Standard care	12'035.13	0.8926	Reference
		Infliximab induction infusions	17'416.83	0.924	171'391.59
		Infliximab maintenance infusions	30'476.26	0.9245	578'091.91
	Moderate active disease refractory to conventional therapies	Standard care	12'035.13	0.8922	Dominated
		Adalimumab induction infusions	8'338.17	0.9231	Reference
		Adalimumab maintenance infusions	21'208.39	0.9236	25'740'443.62
**Ananthakrishnan et al. (2011, USA) [45]**	Patients in surgically-induced remission after first ileocecal resection	Antibiotics arm: Metronidazole given post-operatively. No treatment given if patients experience adverse events on metronidazole unless disease recurred in which case they received infliximab	3'086.90	0.8209	Reference
		Azathioprine arm: Azathioprine given post-operatively. No treatment given if patients experience adverse events on azathioprine unless disease recurred in which case they received infliximab induction and maintenance infusions	3'497.76	0.814	Dominated
		No treatment arm: No treatment given post-operatively. Patients who develop clinical recurrence receive infliximab induction and maintenance infusions	4'265.14	0.805	Dominated
		Tailored infliximab arm: No treatment post-operatively. Patients receive colonoscopy at 6 months; those at no or mild endoscopic recurrence risk received no treatment and those at high endoscopic recurrence risk receive infliximab induction and maintenance infusions	8'728.10	0.8206	Dominated
		Upfront infliximab arm: Infliximab standard dose maintenance infusions given post-operatively. Patients who do not respond to infliximab receive stop treatment and receive no alternative treatment but switch to azathioprine if disease recurs. Patients who develop disease recurrence while on infliximab receive increased infliximab dose (10mg/kg every 8 weeks).	24'070.22	0.828	2'955'396.77
**Ananthakrishnan et al. (2012, USA) [46]**	Moderate to severe disease who lose response to two prior TNF-antagonists	Natalizumab induction and maintenance infusion	56'348.98	0.71	600'858.73
		Certolizumab pegol induction and maintenance injection	50'340.40	0.7	
**Blackhouse et al. (2012, Canada) [47]**	Moderate to severe disease refractory to conventional therapies	Infliximab induction and maintenance infusions	47'928.87	2.721	197'402.17
		Adalimumab induction and maintenance injection	40'304.06	2.701	172'218.88
		Usual care: Immunosuppressants and corticosteroids	15'160.10	2.555	Reference
		Infliximab strategy vs. Adalimumab strategy			360355.43**
**Doherty et al. (2012, USA) [28]**	Patients achieving surgically-induced remission after intestinal resection	Infliximab induction and maintenance infusions	27'311.46	0.87	839'477.61
		Once daily continuous oral azathioprine	7'273.78	0.86	257'332.31
		Once daily continuous oral mesalazine	6'417.28	0.85	Dominated
		No treatment	2'127.14	0.84	Reference
**Tang et al. (2012, USA) [48]**	Moderate to severe disease refractory to conventional therapies and naive to biologic agents	Infliximab induction and maintenance infusions	24'658.25	0.796	Dominant
		Adalimumab induction and maintenance injection	29'957.07	0.799	Dominated
		Certolizumab pegol induction and maintenance injection	31'692.91	0.8	Dominated
		Natalizumab induction and maintenance infusion	33'988.52	0.79	Dominated
**Marchetti et al. (2013, Italy) [49]**§	Moderate to severe newly diagnosed active disease	Top-down arm: Initial induction infusion with infliximab plus azathioprine, followed by infliximab re-treatment and continued azathioprine if symptom exacerbation occurred and finally methylprednisolone added if necessary	20'174.41	3.9	Top-down strategy dominant
		Step up arm: Induction treatment with methylprednisolone, followed by re-treatment with methylprednisolone plus azathioprine if relapse occurred and finally infliximab plus azathioprine added if necessary	21'240.29	3.76	
**Saito et al. (2013, UK) [50]**	Moderate to severe disease refractory to conventional therapies and naive to biologic therapy	Infliximab induction and maintenance infusions plus azathioprine	14'717.04	0.668	4'528.59
		Infliximab induction and maintenance infusions monotherapy	11'981.77	0.064	
**Erim et al. (2015, USA) [51]**	Moderate to severe active disease that failed to respond to infliximab and conventional therapies	Adalimumab and vedolizumab without prior dose increase: Adalimumab induction injections followed by maintenance injections for responders and switch to vedolizumab maintenance infusion for non-responders or patients who lose response	42'065.42	0.83	Reference
		Adalimumab only without dose increase: Adalimumab induction injections and maintenance injections for primary responders	44'229.01	0.81	Dominated
		Adalimumab and vedolizumab with prior dose increase: Adalimumab induction injections followed by maintenance injections for primary responders. For patients who do not respond or lose response receive adalimumab maintenance dose intensification (weekly) or switch to vedolizumab induction and maintenance infusion	45'642.71	0.83	621'851.83
		Adalimumab only with dose increase: Adalimumab induction injection followed by adalimumab maintenance therapy every other week for responders and maintenance therapy weekly for non-responders	48'302.89	0.82	Dominated
**Taleban et al. (2016, USA) [52]**	Medically refractory disease with extensive colitis and no perianal or small bowel inflammation	Total colectomy with ileal pouch anal anastomosis (IPAA)	172'469.72	10.93	Reference
		Total colectomy with permanent end ileostomy (EI)	123'559.09	10.24	70'884.96
**Rafia et al. (2016, UK) Takeda submission [53]**	Moderate to severe active disease after failure of initial therapy	*Mixed population*:			
		Vedolizumab induction and maintenance infusion			Reference
		Conventional therapy (5ASA, immunomodulators, and corticosteroids)			95'213.02
		*Anti-TNF failed population*:			
		Vedolizumab induction and maintenance infusion			Reference
		Conventional therapy (5ASA, immunomodulators, and corticosteroids)			149'021.70
		*Anti-TNF naive population*:			
		Vedolizumab induction and maintenance infusion			Reference
		Conventional therapy (5ASA, immunomodulators, and corticosteroids)			34'387.06
		Infliximab induction and maintenance infusion			40'232.77
		Adalimumab induction and maintenance injection			1'147'866.07

* Conventional therapy/standard of care is defined as drug treatment with aminosalicylates, methotrexate, corticosteroids, azathioprine, metronidazole or surgery; standard dosing approved by FDA and EMA applies unless otherwise specified.

† Unless otherwise stated, the ICER reports the cost per QALY gained

‡ When only ICERs were reported these were converted to 2015 PPP values using the PPP exchange rate for the original currency

§ Year of cost data collection not reported therefore year of publication used to complete PPP conversion

** The indication in this study is “moderate to severe IBD” however, efficacy data was extracted from studies on CD therefore it is assumed that this model reflects the cost-effectiveness for patients with CD. This lack of clarity is captured in the risk of bias assessment.

#### Moderate or severe CD

Priest et al. (2006) showed that maintenance therapy using azathioprine was dominant compared to methotrexate for patients with moderate to severe CD due to lower costs of treatment, fewer adverse events, more patients in remission and increased QALYs [39]. In addition, using first-line infliximab plus azathioprine to induce remission (a top-down strategy) in newly diagnosed patients with moderate to severe CD was dominant compared to the standard step-up approach [49].

Compared to standard care, adalimumab induction and maintenance therapy was cost-effective for severe CD (29’215.03 PPP/QALY) but not for moderate CD (61’323.23 PPP/QALY) in the UK [42]. Additionally, in a lifetime model, infliximab and adalimumab induction and maintenance therapy were cost-effective compared to standard care when maintenance therapy was administered for one or two years only [41]. In these studies, induction and maintenance treatment using adalimumab was cheaper and produced better outcomes compared to infliximab infusions [33, 41, 43].

In a study performed in the USA, for patients who lost response to initial infliximab infusions, switching to adalimumab induction and maintenance therapy was associated with reduced costs and QALYs compared to increasing the infliximab dose to 10mg/kg; however, neither strategy was cost-effective (403’359.61 PPP/QALY) [32]. Alternatively, certolizumab pegol was shown to be a cost-effective third-line biologic agents when compared to natalizumab for induction and maintenance of remission in patients who fail anti-TNF treatment [46].

#### CD refractory to conventional therapies

For patients with CD refractory to conventional treatments, infliximab induction and maintenance therapy was not cost-effective compared to continued treatment with standard care; ICERs ranged from 122’674.42 PPP/QALY to 768’704.19 PPP/QALY in European and Canadian healthcare settings [36–38, 47]. Adalimumab induction and maintenance treatment was also not cost-effective at 172’218.88 PPP/QALY [47]. However, when considering induction doses only, infliximab and adalimumab were dominant compared to standard care for patients with severe disease and adalimumab was cost-effective for patients with moderate disease [44]. ICERs for maintenance treatment strategies, as opposed to induction only and episodic re-treatment (i.e. induce remission, stop treatment and then re-treat when disease recurs), were very high for both infliximab [36–38, 44] and adalimumab [44].

Comparing biologic agents to each other, infliximab induction and maintenance infusions were dominant when compared to adalimumab, certolizumab pegol and natalizumab for patients naive to biologic treatment and refractory to conventional therapies [48] and cost-effective compared to vedolizumab [53]. For patients who failed to respond to infliximab, adalimumab and standard care induction treatments, evidence suggested switching to vedolizumab may be less costly and improve outcomes compared to increasing the dose of adalimumab; however, at current prices, this was not cost-effective in the USA at 621'851.83 PPP/QALY [51]. Similarly, in an anti-TNF naive population in the UK, vedolizumab was not cost-effective compared to standard care, infliximab and adalimumab; however, the gross assumptions made in this model still need to be validated [53].

#### Fistulising CD

For patients with fistulising CD, the ICER for infliximab induction and maintenance infusions compared to standard care was 55’265.19 PPP/QALY in a UK study [40] and 513’552.06 PPP/QALY in the USA [35], which is above accepted cost-effectiveness thresholds. Although still not cost-effective, a single infliximab infusion followed by re-treatment if the fistula recurs, was associated with fewer costs per QALY compared to maintenance infliximab infusions (139’534.88 PPP/QALY versus 170’058.14 PPP/QALY) [37].

#### Surgical and post-surgical interventions

Only one study evaluated the cost-effectiveness of surgery [52]. Total colectomy with permanent end ileostomy was found to be cost-effective compared to total colectomy with ileal pouch-anal anastomosis (IPAA), despite increased QALYs from IPAA, in male patients with isolated medically refractory colonic CD [52]. To maintain remission post-operatively, maintenance treatment with daily azathioprine was cost-effective compared to infliximab maintenance infusions, mesalazine maintenance treatment and no maintenance therapy over a 1 year time horizon [28]. Alternatively, immediate use of antibiotics was the most cost-effective strategy compared to (a) no post-operative treatment, (b) treatment with azathioprine, (c) infliximab infusions for patients at risk of endoscopic recurrence given 6 months after surgery, and (d) immediate post-surgical infliximab infusions [45].

### Ulcerative colitis

The results of the 24 studies on UC are summarised in Table 3.

**Table 3:** Summary of cost-effectiveness results adjusted to 2015 PPP for studies on ulcerative colitis.

Reference (year, country)	Clinical presentation	Interventions & comparators*	Cost (2015 PPP)	Outcome (QALY unless otherwise stated)	ICER (PPP per outcome gained)†
**Panes et al. (2007, Spain) [31]**	Active and steroid-dependent moderate to severe disease	Induction treatment with prednisone followed by 5-ASA maintenance therapy for patients in remission or azathioprine for non-responders	11'236.97	38.50% achieved remission	44'320.62 per remission achieved
		Induction treatment with prednisolone followed by 5-ASA maintenance therapy for patients in remission or granulocyte, monocyte adsorption (GMA)-apheresis for non-responders	21'209.11	61% achieved remission	Reference
**Buckland et al. (2008, UK) [54]**	First line treatment for moderately active disease	Induction therapy using high dose mesalazine (4.8g/day)	4'236.30	0.1394	High dose dominant
		Induction therapy using standard dose mesalazine (2.4g/day)	4'399.92	0.1378	Reference
**Tsai et al. (2008, UK) [55]**	Moderate-severe chronic disease refractory to conventional therapies responding to initial infliximab induction infusions	Maintenance infliximab infusions	120'915.32	4.591	49'922.73
		Standard care	83'323.50	3.838	Reference
	Moderate-severe chronic disease refractory to conventional therapies in remission after initial infliximab induction infusions	Maintenance infliximab infusions	98'016.73	4.154	35'799.74
		Standard care	84'162.23	3.767	Reference
**Yen et al. (2008, USA) [56]**	Mild to moderate disease in remission	No maintenance 5ASA: 5-ASA 4.8g/day given during a flare and stopped once remission achieved	4'145.68	1.75	291'540.46
		Maintenance 5ASA: 5-ASA 2.4g/day given for maintenance treatment and escalated to 4.8g/day after first flare to induce and maintain remission	9'976.49	1.77	Reference
**Connolly et al. (2009a, UK) [57]**	Mild to moderate disease in remission	Once daily 2g mesalazine maintenance therapy	2'011.20	0.935	Once daily mesalazine is dominant
		Twice daily 1g mesalazine maintenance therapy	2'396.16	0.931	Reference
**Connolly et al. (2009b, UK) [58]**	Mild to moderate active disease	Induction treatment with 1g/100ml topical mesalazine plus 4g oral mesalazine combination	4'316.14	0.56	Combination therapy dominant
		Induction treatment with 4g oral mesalazine monotherapy	5'692.92	0.55	Reference
**Xie et al. (2009, Canada) [59]**	Moderate to severe disease refractory to conventional therapies	Strategy A: Standard care (5-ASA or immunosuppressants)	21'506.13	2.015	Reference
		Strategy B: Infliximab induction infusions followed by infliximab maintenance infusions if patient responds. If no response or response lost during maintenance therapy, then switch to adalimumab induction and maintenance injections. If still no response or if response is lost switch to surgery.	73'337.79	2.178	317'985.64
		Strategy C: Infliximab induction infusions followed by infliximab maintenance infusions if patient responds. If no response, escalate dose to 10mg/kg infliximab maintenance infusions. If still no response or response is lost switch to adalimumab induction and maintenance injections	89'746.54	2.149	509'256.80
**Brereton et al. (2010, UK) [60]**	Newly diagnosed or relapsing active mild to moderate disease	5 year model: Induction and maintenance treatment with MMX mesalazine (1200mg tablets once a day)	9'582.42	3.445	1'248.48
		5 year model: Induction and maintenance treatment with Mesalazine (400mg tablets two to three times a day)	9'568.69	3.434	Reference
	Newly diagnosed or relapsing active mild to moderate disease	Lifetime model: Induction and maintenance treatment with MMX Mesalazine (1200mg tablets once a day)	37'196.70	14.861	12'897.00
		Lifetime model: Induction and maintenance treatment with Mesalazine (400mg tablets two to three times a day)	36'693.72	14.822	Reference
**Punekar et al. (2010, UK) [61]**	Patients hospitalised with acute severe exacerbations refractory to intravenous (IV) hydrocortisone	IV cyclosporine plus IV hydrocortisone. If patient responds, switch to oral cyclosporine plus oral prednisolone and azathioprine. For non-responders, switch to surgery	32'970.62	0.7	Reference
		Colectomy: 71% of patients receive illeostomy and 29% of patients receive ileal pouch anal anastomosis (IPAA)	31'051.18	0.58	15'995.29
		Standard care: Continue IV hydrocortisone for 7 days. If patient responds, switch to oral prednisolone and azathioprine. For non-responders, switch to surgery.	33'702.01	0.68	Dominated
		Infliximab induction infusions plus IV hydrocortisone. If patient responds, receive two more infliximab infusions plus prednisolone and azathioprine. For non-responders, switch to surgery	36'109.03	0.8	31'384.13
**Prenzler et al. (2011, Germany) [62]**	Newly diagnosed or relapsing mild to moderate active disease	MMX mesalazine (2400mg/day) induction and maintenance therapy for patients who respond. For non-responders, increase dose to 4800mg/day and if still no response add oral corticosteroids. If still no response or relapse, patient receives immunosuppressants and/or IV steroids and surgery if medical treatment continues to fail.	6'902.31	3.32	MMX is dominant
		Mesalazine (2400mg/day) induction and maintenance therapy for patients who respond. For non-responders, increase dose to 4800mg/day and if still no response add oral corticosteroids. If still no response or relapse, patient receives immunosuppressants and/or IV steroids and surgery if medical treatment continues to fail.	7'774.18	3.309	Reference
**Connolly et al. (2012, Netherlands) [63]**	Mild to moderately active disease	Induction treatment with 1g topical mesalazine combined with 4g oral mesalazine	2'989.80	0.56	Combination therapy is dominant
		Induction treatment with 4g oral mesalazine and placebo enema monotherapy	3'989.56	0.55	Reference
	Mild to moderate disease in remission	Maintenance treatment with once daily 2g mesalazine	1'751.61	0.931	Once daily mesalazine is dominant
		Maintenance treatment with twice daily 1g mesalazine	2'034.74	0.927	Reference
**Park et al. (2012, USA) [29]**	Hospitalised patients with severe pancolitis	Standard medical therapy: IV methylprednisolone followed by mesalazine maintenance treatment for responders; if response lost during maintenance therapy switch to azathioprine. For methylprednisolone non-responders switch to infliximab induction infusions and maintenance infusions for responders. For infliximab non-responders, switch to tacrolimus. If all medical therapies fail, switch to colectomy with IPAA.	261'132.75	20.78	1'631'495.11
		Early colectomy with IPAA: Subtotal colectomy and laparoscopic IPAA given after initial hospitalisation followed by medical treatment for patients with acute or chronic pouchitis.	163'243.05	20.72	Reference
**Saini et al. (2012, USA) [64]**	Recently diagnosed, mild to moderate 5-ASA responsive disease in remission	Inflammation-targeted treatment: patients receive predictive stool testing every 3 months and those with positive test treated with 3-month course of 5-ASA	25'186.38	4.5	Reference
		Symptom-targeted treatment: 5-ASA used for symptomatic disease flares	26'931.90	4.5	623'401.80
		Continuous maintenance treatment: 5-ASA maintenance therapy for all patients in remission	28'305.12	4.5	Dominated
**Chaudhary et al. (2013, Netherlands) [65]**	Patients hospitalised with acute severe exacerbations refractory to IV steroids	Infliximab induction infusions followed by infliximab plus azathioprine and oral steroids for responders. Maintenance treatment continued with azathioprine and oral steroids for responders. Non-responders or patients who lose response switch to surgery.	23'113.73	0.8	Reference
		IV cyclosporine followed by oral cyclosporine plus azathioprine and oral steroids for responders. Maintenance treatment continued with azathioprine and oral steroids for responders. Non-responders or patients who lose response switch to surgery.	20'027.74	0.7	30'859.85
		Surgery with no concomitant medication use	18'937.22	0.58	18'984.14
**Connolly et al. (2014, Netherlands) [66]**	Mild to moderate active disease	Induction therapy with once daily mesalazine	4'001.12	0.57	Once daily mesalazine is dominant
		Induction therapy with twice daily mesalazine	4'583.78	0.56	Reference
**Essat et al. (2014, UK) Takeda submission [67]‡**	Moderate to severe disease refractory or inadequately responding to conventional therapy and anti-TNF alpha agents	*Whole population (patients who received anti-TNF inhibitor and those who did not)*:			
		Conventional therapies: Combination of aminosalicylates, immunomodulators and corticosteroids			49'122.75
		Surgery: 40% of patients have illeostomy and 60% have subtotal proctocolectomy			Dominated
		Vedolizumab: Induction infusions of vedolizumab followed by maintenance infusions for responders. For non-responders switch to surgery. For patients who discontinue biologic treatment switch to conventional therapy			Reference
	Moderate to severe disease refractory or inadequately responding to conventional therapy and anti-TNF alpha agents	*Anti-TNF alpha naive patients*:			
		Conventional therapies (combination of aminosalicylates, immunomodulators and corticosteroids)			7'172.86
		Surgery: 40% of patients have illeostomy and 60% have subtotal proctocolectomy			Dominated
		Infliximab: Induction infusions of infliximab followed by maintenance infusions for responders. For non-responders switch to surgery. For patients who discontinue biologic treatment switch to conventional therapy			Dominated
		Adalimumab: Induction injections of adalimumab followed by maintenance injections for responders. For non-responders switch to surgery. For patients who discontinue biologic treatment switch to conventional therapy			9’787.08
		Golimumab: Induction injections of golimumab followed by maintenance injections for responders. For non-responders switch to surgery. For patients who discontinue biologic treatment switch to conventional therapy			Dominated
		Vedolizumab: Induction infusions of vedolizumab followed by maintenance infusions for responders. For non-responders switch to surgery. For patients who discontinue biologic treatment switch to conventional therapy			Reference
	Moderate to severe disease refractory or inadequately responding to conventional therapy and anti-TNF alpha agents	*Patients who failed TNF-alpha inhibitors*:			
		Conventional therapies: Combination of aminosalicylates, immunomodulators and corticosteroids			95'892.42
		Surgery: 40% of patients have illeostomy and 60% have subtotal proctocolectomy			Dominated
		Vedolizumab: Induction infusions of vedolizumab followed by maintenance infusions for responders. For non-responders switch to surgery. For patients who discontinue biologic treatment switch to conventional therapy			Reference
**Archer et al. (2016, UK) MSD Submission [68]**	Moderate to severe disease refractory or inadequately responding to conventional therapy	Infliximab induction infusions followed by maintenance infusions for responders. For non-responders, switch to relapse management with IV steroids. For patients who fail IV steroids switch to colectomy.	64'509.13	5.7	57'765.06
		Golimumab induction injections followed by maintenance injections for responders. For non-responders, switch to relapse management with IV steroids. For patients who fail IV steroids switch to colectomy.	45'608.55	5.54	40'518.32
		Adalimumab induction injections followed by maintenance injections for responders. For non-responders, switch to relapse management with IV steroids. For patients who fail IV steroids switch to colectomy.	46'651.89	5.49	Dominated
		Immediate colectomy	22'918.28	4.98	Reference
**Archer et al. (2016, UK) Abbvie Submission [68]**	Moderate to severe disease refractory or inadequately responding to conventional therapy	Adalimumab induction and maintenance injections for patients who respond. For non-responders, dose escalation to 40mg every week and switch to conventional therapies if still no response. For non-responders to conventional treatments, switch to surgery.	112'700.41	5.73	50'730.06
		Conventional therapies: Anti-inflammatory drugs or immunosuppressants). For non-responders, switch to colectomy	75'160.16	4.99	Reference
**Beilman et al. (2016, Canada) [69]**	Moderate to severe active corticosteroid-dependent and/or intolerant to thiopurine treatment	No adalimumab: Patients receive no treatment and remain in chronically unwell state to avoid colectomy	89'881.15	3.154	59'398.07
		Adalimumab therapy: Adalimumab induction injections and maintenance injections for responders. For non-responders, switch to steroid therapy.	99'147.25	3.321	Reference
**Stawowczyk et al. (2016, Poland) [70]**	Moderate to severe disease refractory or not responding conventional therapies and contraindicated for cyclosporine	Public payer perspective: Golimumab and standard care combination induction treatment followed by maintenance treatment for responders. For non-responders, switch to standard care alone and, if failure persists, switch to colectomy. Maintenance treatment with golimumab restricted to 1 year.	53'374.23	19.241	222'355.35
		Public payer perspective: Standard care alone induction and maintenance treatment regardless of response. If disease remains active, switch to colectomy.	26'024.52	19.118	Reference
	Moderate to severe active disease refractory or not responding conventional medical therapies and contraindicated for cyclosporine	Societal perspective: Golimumab and standard care combination induction treatment followed by maintenance treatment for responders. For non-responders, switch to standard care alone and colectomy if failure persists. Maintenance treatment with golimumab restricted to 1 year.	173'211.58	19.241	212'762.53
		Societal perspective: Standard care alone, induction and maintenance treatment regardless of response. If disease remains active, switch to colectomy.	147'041.79	19.118	Reference
**Stawowczyk et al. (2016, Poland) [71]**	Moderate to severe active disease refractory to conventional medical therapies	Public payer perspective: Adalimumab and standard care combination induction treatment followed by maintenance treatment for responders. For non-responders, switch to standard care alone and colectomy if failure persists. Maintenance treatment with golimumab restricted to 1 year.	27'464.00	15.204	101'409.52
		Public payer perspective: Standard care alone induction and maintenance treatment regardless of response. If disease remains active, switch to colectomy.	13'266.67	15.064	Reference
		Societal perspective: Adalimumab and standard care combination induction treatment followed by maintenance treatment for responders. For non-responders, switch to standard care alone and colectomy if failure persists. Maintenance treatment with golimumab restricted to 1 year.	125'020.00	15.204	95'190.48
		Societal perspective: Standard care alone induction and maintenance treatment regardless of response. If disease remains active, switch to colectomy.	111'693.33	15.064	Reference
**Stawowczyk et al. (2016, Poland) [72]**	Moderate to severe refractory, intolerant or inadequately responding to conventional medical therapies	Infliximab and standard care combination: Infliximab plus standard care induction infusions followed by maintenance therapy for responders. For non-responders, switch to adalimumab induction injections and maintenance injections for responders. For non-responders to adalimumab, switch to conventional therapy alone or colectomy.	56'425.63	14.296	229'015.09
		Standard care alone: Standard care induction and maintenance treatment. If disease remains active, switch to colectomy.	16'806.02	14.123	Reference
**Tappenden et al. (2016, UK) [73]**	Moderate to severe refractory or intolerant to conventional medical therapies	*Patients in whom surgery is an option*:			
		Colectomy	83'011.66	14.71	Reference
		Adalimumab induction injections followed by maintenance injections for responders. For non-responders, switch to conventional therapy.	134'578.97	10.82	Dominated
		Infliximab induction infusions followed by maintenance infusions for responders. For non-responders, switch to conventional therapy.	142'505.70	10.81	Dominated
		Golimumab induction injections followed by maintenance injections for responders. For non-responders, switch to conventional therapy.	132'904.51	10.63	Dominated
		Conventional treatment for induction and maintenance phases (includes 5-ASA, azathioprine, 6-mercaptopurine, prednisolone)	108'610.90	10.47	Dominated
	Moderate to severe refractory or intolerant to conventional medical therapies	*Patients in whom surgery is not an option*:			
		Adalimumab induction injections followed by maintenance injections for responders. For non-responders, switch to conventional therapy.	134'578.97	10.82	74'194.48
		Infliximab induction infusions followed by maintenance infusions for responders. For non-responders, switch to conventional therapy.	142'505.70	10.81	Extendedly dominated
		Golimumab induction injections followed by maintenance injections for responders. For non-responders, switch to conventional therapy.	132'904.51	10.63	Extendedly dominated
		Conventional treatment for induction and maintenance phases (includes 5-ASA, azathioprine, 6-mercaptopurine, prednisolone)	108'610.90	10.47	Reference
**Yokomizo et al. (2016, USA) [34]‡**	Moderate to severe active disease naive to biologic agents	Infliximab 5mg/kg induction and maintenance infusions			99290.01 per MH achieved
		Infliximab 10mg/kg induction and maintenance infusions			123801.38 per MH achieved
		Adalimumab induction and maintenance injections			316757.65 per MH achieved
		Vedolizumab induction and maintenance infusions			302331.36 per MH achieved
**Wilson et al. (2017, UK) [74]**	Moderate to severe active disease refractory, inadequately responding or lost response to conventional medical therapies and who are anti-TNF naive	Vedolizumab induction infusions followed by maintenance infusions for responders. For non-responders, patients who lose response, or patients who discontinue due to adverse events, switch to conventional therapy. If no response to conventional therapy, switch to another combination of conventional therapies or surgery.	202'422.62	14.077	Reference
		Infliximab induction infusions followed by maintenance infusions for responders. For non-responders, patients who lose response, or patients who discontinue due to adverse events, switch to conventional therapy. If no response to conventional therapy, switch to another combination of conventional therapies or surgery.	209'156.89	13.788	Dominated
		Adalimumab induction infusions followed by maintenance infusions for responders. For non-responders, patients who lose response, or patients who discontinue due to adverse events, switch to conventional therapy. If no response to conventional therapy, switch to another combination of conventional therapies or surgery.	197'686.20	13.972	65'565.01
		Golimumab induction infusions followed by maintenance infusions for responders. For non-responders, patients who lose response, or patients who discontinue due to adverse events, switch to conventional therapy. If no response to conventional therapy, switch to another combination of conventional therapies or surgery.	203'018.58	13.809	Dominated

Conventional therapy/standard of care is defined as drug treatment with aminosalicylates, methotrexate, corticosteroids, azathioprine, metronidazole or surgery; standard dosing approved by FDA and EMA applies unless otherwise specified.

†Unless otherwise stated, the ICER reports the cost per QALY gained

‡ When only ICERs were reported these were converted to 2015 PPP values using the PPP exchange rate for the original currency

#### Mild UC

The cost-effectiveness of high dose MMX^TM^ mesalazine, once daily 2g mesalazine and concomitant oral and topical mesalazine compared to standard oral mesalazine for induction and maintenance of remission was demonstrated across various time horizons in different health systems; ICERs were dominant in five European studies [57, 58, 62, 63, 66]. In contrast, in the USA high dose (4.8g/day) maintenance mesalazine was not cost-effective, despite increased QALYs and decreased risk of flares [56]. Interestingly, an inflammation-targeted re-treatment strategy was shown to dominate maintenance treatment with mesalazine even when costs of a predictive stool test every 3-months is taken into account [64].

#### Moderate or severe UC

Only one study evaluated moderate to severe UC eligible for treatment with conventional medications and found high dose mesalazine was dominant when administered over a short 12 week time horizon due to lower costs compared to standard dose mesalazine (5’878.12 PPP/QALY versus 6’105.16 PPP/QALY) [54].

In addition, colectomy soon after diagnosis of severe UC was more cost-effective than first-line medical therapy (methylprednisolone and azathioprine, followed by infliximab induction and maintenance therapy); however, this study used single-centre cost values potentially reducing the generalisability of these results [29].

#### UC refractory to conventional therapies

Compared to standard care, infliximab induction and maintenance therapy was either dominated [67, 73, 74] or had very high ICERs [68, 72] in studies reflecting European health systems. On the other hand, infliximab was cost-effective for patients hospitalised with acute severe exacerbations and refractory to IV steroids compared to continued IV cyclosporine (30’859.85 PPP/QALY) and surgery (18’984.14 PPP/QALY) [65]. These results support the findings from a similar modelling study based in the UK [61].

Moreover, induction and maintenance treatment with adalimumab produced high ICERs, ranging from 74,194.48 PPP/QALY in the UK to 317,985.64 PPP/QALY in Canada, compared to standard care [59, 71, 73]. However, adalimumab was cost-effective in a Canadian setting when compared to a strategy without adalimumab, including scenarios with no treatment, treatment with steroids and colectomy [69]. Alternatively, in a lifetime model based in the UK, surgery dominated anti-TNF agents and conventional therapies in a subgroup of patients where surgery was acceptable and feasible [73]. When surgery was not feasible, adalimumab dominated infliximab and golimumab but overall conventional therapies were the most cost-effective treatment option.

Recent studies in the UK point to the cost-effectiveness of vedolizumab in an anti-TNF alpha naive population when compared to infliximab, golimumab, adalimumab and conventional therapies; ICERs for each agent ranged from dominance to 9’787 PPP/QALY [67, 74]. Vedolizumab was associated with the highest QALYs compared to anti-TNF alpha agents over the patient’s lifetime [74]. Findings from the USA contradicted this, suggesting that vedolizumab would only be cost-effective as a first-line treatment if drug costs fell below 2’500 USD [34].

### Risk of bias assessments

On average, 67% and 71% of criteria were fulfilled from the Drummond et al. (1996) checklist and 49% and 55% of criteria were fulfilled from the Phillips et al. (2004) checklist for CD and UC, respectively, representing fair quality (see S3 Table). Studies failed to report details on the methods of synthesis of effectiveness data, the population from which utility values were acquired, and disaggregated cost and resource use data. In addition, only 57% of CD studies and 29% of UC studies declared that there were no potential conflicts of interest from researchers and funding sources. This likely reflects the growing demand for the pharmaceutical industry to show not only the clinical effectiveness but also the cost-effectiveness of their products [75].

## Discussion

This review found that, in general, biologic agents help to improve outcomes in terms of QALYs and remission rates; however, at current prices they did not provide good value for money in the majority of clinical situations when compared to conventional therapies. In particular, when administered to maintain remission and when compared to current conventional therapies, biologic agents were not cost-effective in both CD and UC. Moreover, the cost-effectiveness of biologic agents compared to each other remains inconclusive, reflecting a major gap in the literature. Importantly, evidence from CD illustrates the potential for biologic agents to be cost-effective if initiated early (as a top-down strategy) and when the patient’s lifetime clinical management is considered. In addition, in UC, high dose mesalazine for mild disease and early surgical intervention for severe and refractory disease showed greater cost-effectiveness compared to standard of care and biologic agents, respectively. These findings, however, should be reviewed within the context of the methodologies used and the health systems represented in the studies.

ICERs for induction and maintenance treatment with infliximab and adalimumab compared to conventional therapies were well above acceptable cost-effectiveness thresholds in CD and UC refractory to conventional therapies [28, 35–38, 45, 47, 53, 59, 67, 68, 73]. In clinical practice maintenance treatment with biologic agents is preferred to intermittent re-treatment strategies due to the potential development of anti-drug antibodies [76]. Several authors extrapolated the costs and effects of maintenance treatment with biologic agents over a long time horizon, which could explain the high costs incurred over time. In contrast, both infliximab and adalimumab were cost-effective for patients with moderate to severe CD when maintenance treatment was limited to one year [40, 41]. Interestingly, when treatment with adalimumab and infliximab was modelled over the patients’ lifetime rather than one or two years, the ICERs were no longer cost-effective [41]. This suggests an opportunity for the cost-effectiveness of biologic agents if short maintenance therapy schedules are defined and adhered to. Alternatively, maintenance therapy with gradual dose intensification or concomitant treatment with immunomodulators have been suggested to reduce the risk of immunogenicity for both CD and UC; however, the clinical- and cost-effectiveness of these strategies need to be validated [77–79].

The cost-effectiveness of front-line induction therapy using infliximab in newly diagnosed CD patients was an important finding [49]. Current treatment guidelines reserve biologic agents as second-line treatment for moderate to severe disease or when conventional treatments fail [5, 6]. However, early management of CD with infliximab reduced the rate of relapse and hospitalisation compared to patients who received upfront steroids [49]. It has been argued that early intervention with biologic agents in patients who are at high risk of complications may provide long-lasting benefit and help to alter the clinical course of the disease (Moss, 2015). Stratifying patients based on their risk of complications soon after diagnosis may be one way to ensure the value for money of biologic agents is captured [80].

Recent economic evaluations have compared a broader scope of interventions, including newer biologic agents and surgery. For example, in UC refractory to conventional treatments, one study showed vedolizumab was cost-effective compared to anti-TNF agents [74], while another study found surgery was cost-effective compared to conventional and anti-TNF agents [73]. Such evidence was limited in literature on CD, where only one study, submitted by the manufacturers of vedolizumab, compared adalimumab, infliximab and conventional treatments to vedolizumab [53]. Importantly, this study had a high risk of bias due to the assumptions made in the modelling and because the choice of comparators was not comprehensive. Models which incrementally compare treatment strategies are useful for decision-making since they are in line with routine clinical practice where a broad choice of interventions exists.

An important opportunity for the cost-effectiveness of biologic agents is falling drug prices over time due to the increasing number of biologic agents available on the market and in the development pipeline. Moreover, as patents for older biologic agents expire, biologically similar (biosimilar) versions are entering the market, creating an important opportunity for increasing access and reducing costs. Biosimilars to infliximab have been available for IBD since 2013, in Europe, and 2016, in the USA and several biosimilars to adalimumab are in the pipeline [5]. While biosimilars are not identical in molecular structure to their reference products, they have been shown to have similar safety and efficacy profiles [81]. In addition, biosimilars show promise in reducing costs, with initial research suggesting they enter the market at up to 30% lower cost compared to their reference products [82].

Future research is needed to address the gaps identified in the published literature. Firstly, indirect costs (i.e. non-medical costs incurred by the patient due to their disease such as absence from work) were not taken into account in the majority of studies. Indirect costs have been shown to exceed direct costs because IBD is often diagnosed in adolescence and early adulthood and therefore impacts patients’ during their peak productive years [83]. Secondly, studies relied on utility scores from a few studies associated with a high degree of uncertainty [84–86]. When using secondary data sources, there is a risk of introducing bias when specific disease states used in the economic model do not match those for which the utilities were derived. Moreover, evidence suggests, utility scores vary across geographies due to cultural differences [87]. In several studies the utility scores were found to impact the overall cost-effectiveness results significantly; therefore, these should be accurately captured with large samples from the countries evaluated. Future economic models could also help to identify optimal strategies for the use of biologic agents, including the impact of early adoption, risk stratification and the impact of switching between different agents over time [80].

This study has several strengths including that a broad inclusion criteria allowed for an overall understanding of the commonly evaluated treatments in IBD and their cost-effectiveness across different clinical presentations and health systems. In addition, by inflating and converting costs to a common currency we were able to make more reliable comparisons of results between studies. The review methods were documented a priori and approved by all co-authors in order to limit bias in the selection of studies. This systematic literature review incorporates evidence from newer biologic agents and the large number of studies on UC published in 2016, which the latest review did not capture [88]. In addition, this review differs from previous literature reviews which focus only on biologic agents [88] or were less systematic and focused on specific agents and/or diseases [89, 90]. One limitation of the review methods is that one reviewer conducted the literature search, study selection, data extraction and risk of bias assessments, which may have introduced bias into the selection and critical appraisal of studies.

Economic evaluations in IBD have become increasingly popular over the last decade due to the growth of therapeutic options from novel and efficacious biologic agents. While the need for and benefit of systematic reviews in economic evaluations has been contested by some authors [91], this review shows that it is an effective tool to gain an understanding of drivers of treatment costs and benefits across countries. The main limitation to systematic reviews of economic evaluations is the lack of consensus around acceptable cost-effectiveness thresholds. Previous reviews used different thresholds including 35’000 Euros/QALY (38’290 USD) [88] and 100’000 USD/QALY [89]. This study found that studies generally concluded that treatments were cost-effective when ICERs were below 50’000 PPP/QALY. Systematic reviews in health economics could become more effective as a decision-making tool for clinicians and policy makers if consensus on methods of synthesis, taking into account variation in costs across countries and health systems, can be established.

## Conclusion

The results of this review have major implications for future research in this field. Biologic agents were associated with ICERs above 100’000 PPP/QALY in the majority of studies for CD and UC; however, their use consistently demonstrated improvements in quality of life and remission rates. In the future, cost-effectiveness of biologic agents may improve as the market price falls and with the introduction of biosimilars [82]. Future economic models need to strengthen existing literature by more accurately reflecting real world treatment pathways, ensuring the chronic and dynamic nature of IBD is captured and accounting for indirect, as well as direct costs, incurred by the health system and the patients.

## Supporting information

S1 Table Full literature search. (PDF)

S2 Table Descriptive information of included studies. (PDF)

S3 Table Results of the risk of bias assessment. (PDF)

S4 Table PRISMA checklist. (PDF)
