# Supplementary material for: A systematic review of cost-effectiveness studies comparing conventional, biological and surgical interventions for inflammatory bowel disease
Source: PLoS One. 2017 Oct 3;12(10):e0185500. doi: 10.1371/journal.pone.0185500 (PMC5626459; doi:10.1371/journal.pone.0185500)
Supplement: S1 Table — (PDF) [file pone.0185500.s001.pdf]

Supplementary information 1: Detailed literature search

Search in Ovid MEDLINE(R) Epub Ahead of Print, In-Process & Other Non-Indexed Citations, Ovid MEDLINE(R) Daily and Ovid EDLINE(R) 1946 to Present

Number	Search terms	Results
1	crohn* disease.ti,ab,kw.	39445
2	ulcerative colitis.ti,ab,kw.	33430
3	inflammatory bowel disease*.ti,ab,jw.	39791
4	1 or 2 or 3	81955
5	cost effectiveness*.ti,ab,kw.	48099
6	cost utility.ti,ab,kw.	3569
7	cost benefit.ti,ab,kw.	8698
8	health economic*.ti,ab,kw.	6165
9	economic evaluation*.ti,ab,kw.	9451
10	5 or 6 or 7 or 8 or 9	65240
11	4 and 10	280
12	limit 11 to English language	259

Search in Embase 1974-November 9 2016

Number	Search terms	Results
1	crohn* disease.ti,ab,kw.	60956
2	ulcerative colitis.ti,ab,kw.	49359
3	inflammatory bowel disease*.ti,ab,jw.	60016
4	1 or 2 or 3	118867
5	cost effectiveness*.ti,ab,kw.	66971
6	cost utility.ti,ab,kw.	5770
7	cost benefit.ti,ab,kw.	13108
8	health economic*.ti,ab,kw.	9738
9	economic evaluation*.ti,ab,kw.	13216
10	5 or 6 or 7 or 8 or 9	91499
11	4 and 10	552
12	limit 11 to English language	519

Search in York Centers for Reviews and Dissemination (CRD) databases: DARE, NHS EED, HTA

Number	Search	Results
1	(cost benefit) OR (cost effectiveness) OR (cost utility) IN DARE, NHSEED, HTA	19213
2	(crohn* disease) OR (ulcerative colitis) OR (inflammatory bowel disease) IN DARE, NHSEED, HTA	584
3	(economic evaluation) IN DARE, NHSEED, HTA	18503
4	#1 OR #3	22049
5	#2 AND #4	152
