# Supplementary material for: A systematic review of cost-effectiveness studies comparing conventional, biological and surgical interventions for inflammatory bowel disease
Source: PLoS One. 2017 Oct 3;12(10):e0185500. doi: 10.1371/journal.pone.0185500 (PMC5626459; doi:10.1371/journal.pone.0185500)
Supplement: S2 Table — (PDF) [file pone.0185500.s002.pdf]

Supplementary information 2: Study descriptive information

Table 1 Descriptive data and original costs extracted from studies on Crohn's Disease

Reference (year, country)	Model type	Perspective	Time horizon	Interventions/Comparators*	Original cost (currency)	ICER (cost per outcome gained)‡†
**Trallori et al. (1997, unclear) (30)**	None	Societal	Lifetime	Maintenance therapy with mesalazine	5077899 (USD)	5015 USD
				No maintenance treatment	4982619 (USD)	
**Arsenau et al. (2001, USA) (50)**	Markov model	Third party payer	1 year	6MP /metronidazole combination	2894 (USD)	Reference
				Initial infliximab infusions plus combination with 6MP/metronidazole if treatment failure	10003 (USD)	355450
				Initial infliximab infusion with episodic reinfusion if treatment failure	10112 (USD)	360900
				6MP/metronidazole followed by infliximab with episodic reinfusion if treatment failure	6664 (USD)	377000
**Marshall et al. (2002, Canada) (44)**	Markov model	Publically-funded health system	1 year	Strategy A: "usual care" immunosuppressants, intravenous corticosteroids and surgery	9940 (CAD)	Strategy A vs. Strategy B: 181201 CAD
				Strategy B: Single infliximab infusion	12702 (CAD)	Strategy C vs. Strategy B: 480111 CAD
				Strategy C: Single infliximab infusion plus reinfusion for patients who relapse	13739 (CAD)	Strategy D vs. Strategy C: 696078 CAD
				Strategy D: Single infliximab infusion plus maintenance infliximab for patients who respond and usual care for patients who do not respond	21597 (CAD)	
**Clark et al. (2003, UK) Schering-Plough model (42)**	Markov model	Publically-funded health system	Lifetime	Episodic infliximab treatment		10400 GBP
				Single infliximab treatment		6700 GBP
				Maintenance infliximab treatment		84400 GBP
				Placebo		Reference
**Clark et al. (2003, UK) Schering-Plough model (42)**	Markov model	Publically-funded health system		Initial treatment with infliximab		12300 GBP
				Initial treatment with infliximab plus retreatment if fistula reopens		96000 GBP
				Initial treatment with infliximab plus maintenance treatment for patients achieving 100% fistula closure		117000 GBP
				Placebo		Reference

**Clark et al. (2003, UK) Primary economic evaluation (42)**	Markov model	Publically-funded health system	Other	Infliximab (5mg/kg) single dose		93244 GBP
				Infliximab (5mg/kg) episodic dose		62016 GBP
				Infliximab (all doses) single dose		135333 GBP
				Infliximab (all doses) episodic		72261 GBP
				Placebo		Reference
**Jaisson-Hot et al. (2004, France) (43)**	Markov model	Third party payer	Lifetime	Strategy 1a: Initial infliximab infusion plus re-treatment when patients relapse or do not respond	119801.60 (Euros)	63700.82 Euros
				Strategy 1b: Initial infliximab infusion plus maintenance infliximab infusions every 8 weeks	687086.96 (Euros)	784057 Euros
				Strategy 2: Surgery	71296.44 (Euros)	Reference
**Priest et al. (2006, NZ) (35)[‡]**	Decision analytic model	Third party payer	1 year	Azathioprine maintenance therapy	972891 (USD)	Azathioprine dominant
				Methotrexate maintenance therapy	1190191 (USD)	
**Kaplan et al. (2007, USA) (32)**	Decision analytic model	Not clear	1 year	Infliximab dose escalation to 10mg/kg every 8 weeks	28367 (USD)	332032 USD
				Discontinue infliximab and switch to adalimumab induction and maintenance therapy	18074 (USD)	
**Lindsay et al. (2008, UK) (49)**	Markov model	Publically-funded health system	5 years	Infliximab initial infusions and maintenance treatment	31499 (GBP)	26128 GBP
				Standard care (immunomodulators and/or corticosteroids)	26627 (GBP)	
**Lindsay et al. (2008, UK) (49)**	Markov model	Publically-funded health system	5 years	Infliximab initial infusions and maintenance therapy	37488 (GBP)	29752 GBP
				Standard care (immunomodulators and/or corticosteroids)	31490 (GBP)	
**Bodger et al. (2009, UK) (37)**	Markov model	Publically-funded health system	Lifetime	Infliximab infusions for induction of remission followed by maintenance treatment for 1 year	50330 (GBP)	19050 GBP
				Infliximab infusions for induction of remission followed by maintenance treatment for 2 years	58230 (GBP	21300 GBP
				Adalimumab injection for induction of remission followed by maintenance treatment for 1 year	46730 (GBP)	7190 GBP
				Adalimumab injection for induction of remission followed by maintenance treatment for 2 year	53090 (GBP)	10310 GBP
				Standard care (5ASA, immunosuppressive agents, corticosteroids, antibiotics, symptomatic therapies, topical therapies and surgery)	43490 (GBP)	Reference

**Loftus et al. (2009, UK) (39)**	Decision analytic model	Publically-funded health system	1 year	Adalimumab maintenance therapy injection	10882 (GBP)	16064 GBP
				Non-biologic therapy (based on the CLASSIC I trial: placebo and conventional medications)	8992 (GBP)	Reference
				Adalimumab maintenance therapy injection	9696 (GBP)	33731 GBP
				Non-biologic therapy (based on the CLASSIC I trial: placebo and conventional medications)	6649 (GBP)	
**Yu et al. (2009, USA) (38)**	Decision analytic model	Third party payer	1 year	Adalimumab maintenance therapy injection	34193 (USD)	Adalimumab dominant
				Infliximab maintenance therapy infusion	39045 (USD)	
**Bakhshai et al. (2010, USA) (33)**	Decision analytic model	Third party payer	2 years	Natalizumab induction and maintenance infusion	68372 (USD)	Reference
				Infliximab induction and maintenance infusions	62090 (USD)	Dominated by adalimumab
				Adalimumab induction and maintenance injection	61796 (USD)	4059.26 per month of remission
**Dretzke et al. (2011, UK) (45)**	Markov model	Publically-funded health system	1 year	Standard care	13415 (GBP)	Dominated
				Infliximab induction infusions	12051 (GBP)	Reference
				Infliximab maintenance infusions	19143 (GBP)	5.03 million GBP
				Standard care	13421 (GBP)	Dominated
				Adalimumab induction infusions	7053 (GBP)	Reference
				Adalimumab maintenance infusions	14047 (GBP)	4.98 million GBP
				Standard care	6615 (GBP)	Reference
				Infliximab induction infusions	9573 (GBP)	94321 GBP
				Infliximab maintenance infusions	16751 (GBP)	13.9 million GBP
				Standard care	6615 (GBP)	Dominated
				Adalimumab induction infusions	4583 (GBP)	Reference
				Adalimumab maintenance infusions	11657 (GBP)	13.9 million GBP
**Ananthakrishnan et al. (2011, USA) (52)**	Decision analytic model	Third party payer	1 year	Antibiotics arm: Metronidazole given post-operatively. No treatment given if patients experience adverse events on metronidazole unless disease recurred in which case they received infliximab	2840 (USD)	Reference
				Azathioprine arm: Azathioprine given post-operatively. No treatment given if patients experience adverse events on azathioprine unless disease recurred in which case they received	3218 (USD)	Dominated

				infliximab induction and maintenance infusions		
				No treatment arm: No treatment given post-operatively. Patients who develop clinical recurrence receive infliximab induction and maintenance infusions	3924 (USD)	Dominated
				Tailored infliximab arm: No treatment post-operatively. Patients receive colonoscopy at 6 months; those at no or mild endoscopic recurrence risk received no treatment and those at high endoscopic recurrence risk receive infliximab induction and maintenance infusions	8030 (USD)	Dominated
				Upfront infliximab arm: Infliximab standard dose maintenance infusions given post-operatively. Patients who do not respond to infliximab receive stop treatment and receive no alternative treatment but switch to azathioprine if disease recurs. Patients who develop disease recurrence while on infliximab receive increased infliximab dose (10mg/kg every 8 weeks).	22145 (USD)	2757857 USD
**Ananthakrishnan et al. (2012, USA) (40)**	Decision analytic model	Third party payer	1 year	Natalizumab induction and maintenance infusion	51842 (USD)	381,678 USD
				Certolizumab pegol induction and maintenance injection	46314 (USD)	
**Blackhouse et al. (2012, Canada) (41)**	Markov model	Publically-funded health system	5 years	Infliximab induction and maintenance infusions	54084 (CAD)	222955 CAD
				Adalimumab induction and maintenance injection	45480 (CAD)	193305 CAD
				Usual care: Immunosuppressants and corticosteroids	17107 (CAD)	Reference
				Infliximab strategy vs. Adalimumab strategy		451165 CAD
**Doherty et al. (2012, USA) (28)**	Monte Carlo simulation	Societal	1 year	Infliximab induction and maintenance infusions	25127 (USD)	831912 USD
				Once daily continuous oral azathioprine	6692 (USD)	299188 USD
				Once daily continuous oral mesalazine	5904 (USD)	Dominated
				No treatment	1957 (USD)	Reference
**Tang et al. (2012, USA) (46)**	Monte Carlo simulation	Third party payer	1 year	Infliximab induction and maintenance infusions	22686 (USD)	Dominant strategy
				Adalimumab induction and maintenance injection	27561 (USD)	Dominated
				Certolizumab pegol induction and maintenance injection	29158 (USD)	Dominated
				Natalizumab induction and maintenance infusion	31270 (USD)	Dominated

**Marchetti et al. (2013, Italy) (36)**	Markov model	Third party payer	5 years	Top-down arm: Initial induction infusion with infliximab plus azathioprine, followed by infliximab re-treatment and continued azathioprine if symptom exacerbation occurred and finally methylprednisolone added if necessary	14631 (Euros)	Top-down strategy dominant
				Step up arm: Induction treatment with methylprednisolone, followed by re-treatment with methylprednisolone plus azathioprine if relapse occurred and finally infliximab plus azathioprine added if necessary	15404 (Euros)	
**Saito et al. (2013, UK) (90)**	Decision analytic model	Publically-funded health system	1 year	Infliximab induction and maintenance infusions plus azathioprine	8573.04 (GBP)	24917 GBP
				Infliximab induction and maintenance infusions monotherapy	6979.68 (GBP)	
**Erim et al. (2015, USA) (48)**	Markov cohort model	Third party payer	1 year	Adalimumab plus vedolizumab without prior dose increase: Adalimumab induction injections followed by maintenance injections for responders and switch to vedolizumab maintenance infusion for non-responders or patients who lose response	42015 (USD)	Reference
				Adalimumab strategy without dose increase: Adalimumab induction injections and maintenance injections for primary responders	44176 (USD)	Dominated
				Adalimumab plus vedolizumab with prior dose increase: Adalimumab induction injections followed by maintenance injections for primary responders. For patients who do not respond or lose response receive adalimumab maintenance dose intensification (weekly) or switch to vedolizumab induction and maintenance infusion	45588 (USD)	611974 USD
				Adalimumab with dose increase: Adalimumab induction injection followed by adalimumab maintenance therapy every other week for responders and maintenance therapy weekly for non-responders	48245 (USD)	Dominated
**Taleban et al. (2016, USA) (51)**	Markov model	Third party payer	Lifetime	Total colectomy with ileal pouch anal anastomosis (IPAA)	172263 (USD)	70715 USD
				Total colectomy with permanent end ileostomy (EI)	123411 (USD)	
**Rafia et al. (2016, UK) Takeda submission (47)**	Markov model	Publically-funded health system	10 year	Mixed population:		
				Vedolizumab induction and maintenance infusion		Reference
				Conventional therapy (5ASA, immunomodulators,		62903 GBP

	and corticosteroids)	
	Anti-TNF failed population:	
	Vedolizumab induction and maintenance infusion	Reference
	Conventional therapy (5ASA, immunomodulators, and corticosteroids)	98452 GBP
	Anti-TNF naive population:	
	Vedolizumab induction and maintenance infusion	Reference
	Conventional therapy (5ASA, immunomodulators, and corticosteroids)	22718 GBP
	Infliximab induction and maintenance infusion	26580 GBP
	Adalimumab induction and maintenance injection	758344 GBP

[*] Conventional therapy/standard of care defined as drug treatment with aminosalicylates, methotrexate, corticosteroids, azathioprine, metronidazole or surgery; standard dosing approved by FDA and EMA applies unless otherwise specified.

[†] Unless otherwise stated, the ICER reports the cost per QALY gained

[‡] The indication in this study is “moderate to severe IBD” however, efficacy data was extracted from studies on CD therefore it is assumed that this model reflects the cost-effectiveness for patients with CD. This lack of clarity is captured in the risk of bias assessment.

Table 2 Descriptive data and original costs extracted from studies on Ulcerative Colitis

Reference (year, country)	Model type	Perspective	Time horizon	Interventions & comparators*	Cost (currency)	ICER (cost per outcome gained)†
**Panes et al. (2007, Spain) (31)**	Decision analytic model	Third party payer	1 year	Induction treatment with prednisone followed by 5-ASA maintenance therapy for patients in remission or azathioprine for non-responders	6059 (Euros)	23898 Euros
				Induction treatment with prednisolone followed by 5-ASA maintenance therapy for patients in remission or granulocyte manocyte adsorptive (GMA)-apheresis for non-responders	11436 (Euros)	
**Buckland et al. (2008, UK) (61)**	Decision analytic model	Publically-funded health system	12 weeks	Induction therapy using high dose mesalazine (4.8g/day)	2382 (GBP)	High dose dominant
				Induction therapy using standard dose mesalazine (2.4g/day)	2474 (GBP)	
**Tsai et al. (2008, UK) (73)**	Markov model	Publically-funded health system	10 years	Patients responding to initial infliximab infusions: Maintenance infliximab infusions	66460 (GBP)	27424 GBP
				Standard care	45798 (GBP)	
				Patients in remission after initial infliximab infusions: Maintenance infliximab infusions	53874 (GBP)	19696 GBP
				Standard care	46259 (GBP)	
**Yen et al. (2008, USA) (60)**	Markov model	Third party payer	2 years	No maintenance 5ASA: 5-ASA 4.8g/day given during a flare and stopped once remission achieved	3304 (USD)	224000 USD
				Maintenance 5ASA: 5-ASA 2.4g/day given for maintenance treatment and escalated to 4.8g/day after first flare to induce and maintain remission	7951 (USD)	
**Connolly et al. (2009a, UK) (57)**	Decision analytic model	Publically-funded health system	1 year	Once daily mesalazine maintenance therapy	815 (GBP)	Once daily mesalazine is dominant
				Twice daily mesalazine maintenance therapy	971 (GBP)	
**Connolly et al. (2009b, UK) (56)**	Markov model	Publically-funded health system	Not stated	Induction treatment with topical mesalazine plus oral mesalazine combination	1812 (GBP)	Combination therapy dominant
				Induction treatment with oral mesalazine monotherapy	2390 (GBP)	
**Xie et al. (2009, Canada) (67)**	Markov model	Publically-funded health system	5 years	Strategy A: Standard care (5-ASA or immunosuppressants)	24268 (CAD)	Reference
				Strategy B: Infliximab induction infusions followed by infliximab maintenance infusions if patient	82756 (CAD)	358088 CAD

				responds. If no response or response lost during maintenance therapy, then switch to adalimumab induction and maintenance injections. If still no response or if response is lost switch to surgery.		
				Strategy C: Infliximab induction infusions followed by infliximab maintenance infusions if patient responds. If no response, escalate dose to 10mg/kg infliximab maintenance infusions. If still no response or response is lost switch to adalimumab induction and maintenance injections	101272 (CAD)	575540 CAD
**Brereton et al. (2010, UK) (54)**	Markov cohort model	Publically-funded health system	5 years	5 year model: Induction and maintenance treatment with MMX mesalazine (1200mg tablets once a day)	5582 (GBP)	749 GBP
				5 year model: Induction and maintenance treatment with Mesalazine (400mg tablets two to three times a day)	5574 (GBP)	
				Lifetime model: Induction and maintenance treatment with MMX Mesalazine (1200mg tablets once a day)	21668 (GBP)	7600 GBP
				Lifetime model: Induction and maintenance treatment with Mesalazine (400mg tablets two to three times a day)	21375 (GBP)	
**Punekar et al. (2010, UK) (71)**	Decision analytic model	Publically-funded health system	1 year	Cyclosporine: IV cyclosporine plus IV hydrocortisone. If patient responds, switch to oral cyclosporine plus oral prednisolone and azathioprine. For non-responders, switch to surgery	18122 (GBP)	Reference
				Colectomy: 71% of patients receive illeostomy and 29% of patients receive ileal pouch anal anastomosis (IPAA)	17067 (GBP)	9,032 GBP
				Standard care: Continue IV hydrocortisone for 7 days. If patient responds, switch to oral prednisolone and azathioprine. For non-responders, switch to surgery.	18524 (GBP)	Dominated
				Infliximab: Infliximab induction infusions plus IV hydrocortisone. If patient responds, receive two more infliximab infusions plus prednisolone and azathioprine. For non-responders, switch to surgery	19847 (GBP)	18388 GBP
**Prenzler et al. (2011, Germany) (53)**	Markov model	Third party payer	5 years	MMX mesalazine (2400mg/day) induction and maintenance therapy for patients who respond. For non-responders, increase dose to 4800mg/day and if still no response add oral corticosteroids. If still no response or relapse,	4940 (Euros)	MMX is dominant

				patient receives immunosuppressants and/or IV steroids and surgery if medical treatment continues to fail.		
				Mesalazine (2400mg/day) induction and maintenance therapy for patients who respond. For non-responders, increase dose to 4800mg/day and if still no response add oral corticosteroids. If still no response or relapse, patient receives immunosuppressants and/or IV steroids and surgery if medical treatment continues to fail.	5564 (Euros)	
**Connolly et al. (2012, Netherlands) (55)**	Decision analytic model	Publically-funded health system	1 year	Induction treatment with topical mesalazine combined with oral mesalazine	2207 (Euros)	Combination therapy is dominant
				Induction treatment with oral mesalazine monotherapy	2945 (Euros)	
				Maintenance treatment with once daily mesalazine	1293 (Euros)	Once daily mesalazine is dominant
				Maintenance treatment with twice daily mesalazine	1502 (Euros)	
**Park et al. (2012, USA) (29)**	Markov model	Societal	Lifetime	Standard medical therapy: IV methylprednisolone followed by mesalazine maintenance treatment for responders; if response lost during maintenance therapy switch to azathioprine. For methylprednisolone non-responders switch to infliximab induction infusions and maintenance infusions for responders. For infliximab non-responders, switch to tacrolimus. If all medical therapies fail, switch to colectomy with IPAA.	236370 (USD)	1476783 USD
				Early colectomy with IPAA: Subtotal colectomy and laparoscopic IPAA given after initial hospitalisation followed by medical treatment for patients with acute or chronic pouchitis.	147763 (USD)	
**Saini et al. (2012, USA) (59)**	Markov cohort model	Third party payer	5 years	Inflammation-targeted treatment: Patients receive predictive stool testing every 3 months and those with positive test treated with 3-month course of 5-ASA	22798 (USD)	Reference
				Symptom-targeted treatment: 5-ASA used for symptomatic disease flares	24378 (USD)	575894 USD
				Continuous maintenance treatment: 5-ASA maintenance therapy for all patients in remission	25621 (USD)	Dominated
**Chaudhary et al. (2013, Netherlands)**	Markov model	Third party payer	1 year	Infliximab induction infusions followed by infliximab plus azathioprine and oral steroids for	17062 (Euros)	Reference

**(70)**				responders. Maintenance treatment continued with azathioprine and oral steroids for responders.Non-responders or patients who lose response switch to surgery.		
				IV cyclosporine followed by oral cyclosporine plus azathioprine and oral steroids for responders. Maintenance treatment continued with azathioprine and oral steroids for responders. Non-responders or patients who lose response switch to surgery.	14784 (Euros)	24277 Euro
				Surgery with no concomitant medication use	13979 (Euros)	14639 Euro
**Connolly et al. (2014, Netherlands) (58)**	Markov model	Publically-funded health system	32 weeks	Induction therapy with once daily mesalazine	3097 (Euros)	Once daily mesalazine is dominant
				Induction therapy with twice daily mesalazine	3548 (Euros)	
**Essat et al. (2014, UK) Takeda submission (62)**	Markov model	Publically-funded health system	10 years	*Whole population (patients who received anti-TNF inhibitor and those who did not):*		
				Conventional therapies: Combination of aminosalicylates, immunomodulators and corticosteroids	Unknown	33297 GBP
				Surgery: 40% of patients have illeostomy and 60% have subtotal proctocolectomy		Dominated
				Vedolizumab: Induction infusions of vedolizumab followed by maintenance infusions for responders. For non-responders switch to surgery. For patients who discontinue biologic treatment switch to conventional therapy		Reference
				Anti-TNF alpha naive patients:		
				Conventional therapies (combination of aminosalicylates, immunomodulators and corticosteroids)	Unknown	4862 GBP
				Surgery: 40% of patients have illeostomy and 60% have subtotal proctocolectomy		Dominated
				Infliximab: Induction infusions of infliximab followed by maintenance infusions for responders. For non-responders switch to surgery. For patients who discontinue biologic treatment switch to conventional therapy		Dominated
				Adalimumab: Induction injections of adalimumab followed by maintenance injections for responders. For non-responders switch to surgery. For patients who discontinue biologic treatment		66634 GBP

				switch to conventional therapy		
				Golimumab: Induction injections of golimumab followed by maintenance injections for responders. For non-responders switch to surgery. For patients who discontinue biologic treatment switch to conventional therapy		Dominated
				Vedolizumab: Induction infusions of vedolizumab followed by maintenance infusions for responders. For non-responders switch to surgery. For patients who discontinue biologic treatment switch to conventional therapy		Reference
				Patients who failed TNF-alpha inhibitors:		
				Conventional therapies: Combination of aminosalicylates, immunomodulators and corticosteroids	Unknown	64999 GBP
				Surgery: 40% of patients have illeostomy and 60% have subtotal proctocolectomy		Dominated
				Vedolizumab: Induction infusions of vedolizumab followed by maintenance infusions for responders. For non-responders switch to surgery. For patients who discontinue biologic treatment switch to conventional therapy		Reference
**Archer et al. (2016, UK) MSD Submission (65)**	Markov model	Publically-funded health system	10 years	Infliximab induction infusions followed by maintenance infusions for responders. For non-responders, switch to relapse management with IV steroids. For patients who fail IV steroids switch to colectomy.	44382.28 (GBP)	80316 GBP
				Golimumab induction injections followed by maintenance injections for responders. For non-responders, switch to relapse management with IV steroids. For patients who fail IV steroids switch to colectomy.	31378.68 (GBP)	27994 GBP
				Adalimumab induction injections followed by maintenance injections for responders. For non-responders, switch to relapse management with IV steroids. For patients who fail IV steroids switch to colectomy.	32096.50 (GBP)	Dominated
				Immediate colectomy	15767.78 (GBP)	Reference
**Archer et al. (2016, UK) Abbvie Submission (65)**	Markov model	Publically-funded health system	10 years	Adalimumab induction and maintenance injections for patients who respond. For non-responders, dose escalation to 40mg every week and switch to conventional therapies if still no response. For non-responders to conventional treatments, switch	76392 (GBP)	34417 GBP

				to surgery.		
				Conventional therapies: Anti-inflammatory drugs or immunosuppressants). For non-responders, switch to colectomy	50946 (GBP)	
**Beilman et al. (2016, Canada) (69)**	Markov model	Publically-funded health system	10 years	No adalimumab: Patients receive no treatment and remain in chronically unwell state to avoid colectomy	97000 (CAD)	59000 CAD
				Adalimumab therapy: Adalimumab induction injections and maintenance injections for responders. For non-responders, switch to steroid therapy.	107000 (CAD)	
**Stawowczyk et al. (2016, Poland) (85)**	Markov model	Societal	Lifetime	Public payer perspective: Golimumab and standard care combination induction treatment followed by maintenance treatment for responders. For non-responders, switch to standard care alone and colectomy if failure persists. Maintenance treatment with golimumab restricted to 1 year.	93321 (PLN)	391252 PLN
				Public payer perspective: Standard care alone induction and maintenance treatment regardless of response. If disease remains active, switch to colectomy.	45502 (PLN)	
				Societal perspective: Golimumab and standard care combination induction treatment followed by maintenance treatment for responders. For non-responders, switch to standard care alone and colectomy if failure persists. Maintenance treatment with golimumab restricted to 1 year.	302848 (PLN)	374377 PLN
				Societal perspective: Standard care alone induction and maintenance treatment regardless of response. If disease remains active, switch to colectomy.	257092 (PLN)	
**Stawowczyk et al. (2016, Poland) (68)**	Markov model	Societal	Lifetime	Public payer perspective: Adalimumab and standard care combination induction treatment followed by maintenance treatment for responders. For non-responders, switch to standard care alone and colectomy if failure persists. Maintenance treatment with golimumab restricted to 1 year.	20598 (Euros)	76120 Euros
				Public payer perspective: Standard care alone induction and maintenance treatment regardless of response. If disease remains active, switch to colectomy.	9950 (Euros)	
				Societal perspective: Adalimumab and standard	93765 (Euros)	71457 Euros

				care combination induction treatment followed by maintenance treatment for responders. For non-responders, switch to standard care alone and colectomy if failure persists. Maintenance treatment with golimumab restricted to 1 year.		
				Societal perspective: Standard care alone induction and maintenance treatment regardless of response. If disease remains active, switch to colectomy.	83770 (Euros)	
**Stawowczyk et al. (2016, Poland) (66)**	Markov model	Societal	Lifetime	Infliximab and standard care combination: Infliximab plus standard care induction infusions followed by maintenance therapy for responders. For non-responders, switch to adalimumab induction injections and maintenance injections for responders. For non-responders to adalimumab, switch to conventional therapy alone or colectomy.	99522 (PLN)	402420 PLN
				Standard care alone: Standard care induction and maintenance treatment. If disease remains active, switch to colectomy.	29642 (PLN)	
**Tappenden et al. (2016, UK) (63)**	Markov model	Publically-funded health system	Lifetime	*Patients in whom surgery is an option:*		
				Colectomy	56268 (GBP)	Reference
				Adalimumab induction injections followed by maintenance injections for responders. For non-responders, switch to conventional therapy.	91222 (GBP)	Dominated
				Infliximab induction infusions followed by maintenance infusions for responders. For non-responders, switch to conventional therapy.	96595 (GBP)	Dominated
				Golimumab induction injections followed by maintenance injections for responders. For non-responders, switch to conventional therapy.	90087 (GBP)	Dominated
				Conventional treatment for induction and maintenance phases (includes 5-asas, azathioprine, 6-mercaptopurine, prednisolone)	73620 (GBP)	Dominated
				Patients in whom surgery is not an option:		
				Adalimumab induction injections followed by maintenance injections for responders. For non-responders, switch to conventional therapy.	91222 (GBP)	50728 GBP
				Infliximab induction infusions followed by maintenance infusions for responders. For non-responders, switch to conventional therapy.	96595 (GBP)	Extendedly dominated
				Golimumab induction injections followed by	90087 (GBP)	Extendedly dominated

				maintenance injections for responders. For non-responders, switch to conventional therapy.		
				Conventional treatment for induction and maintenance phases (includes 5-asas, azathioprine, 6-mercaptopurine, prednisolone)	73620 (GBP)	Reference
**Yokomizo et al. (2016, USA) (34)**	Decision analytic model	Third party payer	1 year	Infliximab 5mg/kg induction and maintenance infusions	Unknown	99171 USD per MH achieved
				Infliximab 10mg/kg induction and maintenance infusions		123653 USD per MH achieved
				Adalimumab induction and maintenance injections		316378 USD per MH achieved
				Vedolizumab induction and maintenance infusions		301969 USD per MH achieved
**Wilson et al. (2017, UK) (64)**	Markov model	Publically funded health system	Lifetime	Vedolizumab induction infusions followed by maintenance infusions for responders. For non-responders, patients who lose response, or patients who discontinue due to adverse events, switch to conventional therapy. If no response to conventional therapy, switch to another combination of conventional therapies or surgery.	199431.15 GBP	Reference
				Infliximab induction infusions followed by maintenance infusions for responders. For non-responders, patients who lose response, or patients who discontinue due to adverse events, switch to conventional therapy. If no response to conventional therapy, switch to another combination of conventional therapies or surgery.	206065.90 GBP	Dominated
				Adalimumab induction infusions followed by maintenance infusions for responders. For non-responders, patients who lose response, or patients who discontinue due to adverse events, switch to conventional therapy. If no response to conventional therapy, switch to another combination of conventional therapies or surgery.	194764.73 GBP	22775 GBP

	Golimumab induction infusions followed by maintenance infusions for responders. For non-responders, patients who lose response, or patients who discontinue due to adverse events, switch to conventional therapy. If no response to conventional therapy, switch to another combination of conventional therapies or surgery.	200018.31 GBP	Dominated

---

* Conventional therapy/standard of care defined as drug treatment with aminosalicylates, methotrexate, corticosteroids, azathioprine, metronidazole or surgery; standard dosing approved by FDA and EMA applies unless otherwise specified.

† Unless otherwise stated, the ICER reports the cost per QALY gained
