# Supplementary material for: A systematic review of cost-effectiveness studies comparing conventional, biological and surgical interventions for inflammatory bowel disease
Source: PLoS One. 2017 Oct 3;12(10):e0185500. doi: 10.1371/journal.pone.0185500 (PMC5626459; doi:10.1371/journal.pone.0185500)
Supplement: S3 Table — (PDF) [file pone.0185500.s003.pdf]

Supplementary Information 3: Risk of bias assessments

Table 1 Risk of bias assessment for studies on Crohn's Disease

	Reference	Tralori (1997)	Arsenau et al. (2001)	Marshall (2002)	Clark et al. (description of manufacturers chronic acive model) (2003)	Clark et al. (description of manufacturers fistulising model) (2003)	Clark et al. (Reassess manufacturer's model (2003)	Jaisson-Hot et al. (2004)	Priest et al. (2006)	Kaplan et al. (2007)	Lindsay et al. (2008)	Bodger et al. (2009)	Loftus et al. (2009)	Yu et al. (2009)	Bakhshai et al. (2010)	Ananthakrishnan et al. (2011)	Dretzke et al. (2011)	Ananthakrishnan et al. (2012)	Blackhouse et al. (2012)	Doherty et al. (2012)	Tang et al. (2012)	Marchetti et al. (2013)	Saito et al. (2013)	Erim et al. (2015)	Rafia (2016)	Taleban et al. (2016)
	Drummond et al. (1996) checklist																									
	Study design																									
1	The research question is stated.	Y	Y	Y	Y	Y	Y	Y	Y	Y	Y	Y	Y	Y	Y	Y	Y	Y	Y	Y	Y	Y	Y	Y	Y	Y
2	The economic importance of the research question is stated.	Y	Y	Y	Y	Y	Y	Y	Y	Y	Y	Y	Y	Y	Y	Y	Y	Y	Y	Y	Y	Y	Y	Y	Y	Y
3	The viewpoint(s) of the analysis are clearly stated and justified.	N	Y	Y	Y	Y	Y	Y	Y	N	Y	Y	Y	Y	Y	Y	Y	Y	Y	Y	Y	Y	Y	Y	Y	Y
4	The rationale for choosing alternative programmes or interventions compared is stated.	Y	Y	Y	N	N	N	Y	Y	Y	Y	Y	Y	Y	Y	Y	Y	Y	Y	Y	Y	Y	Y	Y	Y	Y
5	The alternatives being	N	Y	Y	Y	Y	Y	Y	Y	Y	Y	Y	Y	Y	Y	Y	Y	Y	Y	Y	Y	Y	Y	Y	Y	Y

	compared are clearly described.																									
6	The form of economic evaluation used is stated.	Y	Y	Y	Y	Y	Y	Y	Y	Y	Y	Y	Y	Y	Y	Y	Y	Y	Y	Y	Y	Y	Y	Y	Y	Y
7	The choice of form of economic evaluation is justified in relation to the questions addressed.	Y	Y	Y	N	N	N	Y	N	Y	Y	Y	Y	Y	N	Y	Y	Y	Y	Y	Y	Y	Y	Y	Y	Y
	Data collection																									
8	The source(s) of effectiveness estimates used are stated.	N	Y	Y	Y	Y	Y	Y	Y	Y	Y	Y	Y	Y	Y	Y	Y	Y	Y	Y	Y	Y	Y	Y	Y	Y
9	Details of the design and results of effectiveness study are given (if based on a single study).	N	NA	N	NA	NA	N	Y	NA	NA	NA	NA	NA	NA	NA	NA	NA	NA	NA	NA	NA	Y	NA	NA	NA	NA
10	Details of the methods of synthesis or meta-analysis of estimates are given (if based on a synthesis of a number of effectiveness studies).	N	Y	NA	N	N	NA	NA	N	N	N	Y	N	Y	N	N	Y	N	N	N	N	NA	N	N	Y	N
11	The primary outcome measure(s) for the economic evaluation are clearly stated.	Y	Y	Y	Y	Y	Y	Y	Y	Y	Y	Y	Y	Y	Y	Y	Y	Y	Y	Y	Y	Y	Y	Y	Y	Y
12	Methods to value benefits are stated.	Y	Y	Y	Y	N	N	Y	Y	Y	Y	Y	Y	Y	N	Y	Y	Y	Y	Y	Y	Y	N	Y	Y	Y
13	Details of the subjects from whom valuations were obtained were given.	N	Y	N	Y	N	N	Y	Y	N	N	N	Y	Y	N	N	N	N	N	N	N	N	N	N	N	N
14	Productivity changes (if included) are reported separately.	NA	NA	NA	NA	NA	NA	NA	NA	NA	NA	NA	Y	NA	NA	NA	NA	NA	NA	N	NA	NA	NA	NA	NA	NA
15	The relevance of productivity changes to the study question is discussed.	N	Y	Y	N	N	N	Y	N	N	Y	N	Y	Y	N	N	N	N	Y	N	N	Y	N	Y	N	N
16	Quantities of resource use are reported separately from their unit costs.	N	Y	N	N	N	N	N	N	N	Y	N	N	Y	N	N	Y	N	Y	N	N	Y	N	N	N	N

17	Methods for the estimation of quantities and unit costs are described.	Y	Y	Y	N	N	Y	Y	Y	Y	Y	Y	Y	Y	N	Y	Y	Y	Y	Y	Y	Y	Y	Y	Y	Y
18	Currency and price data are recorded.	Y	Y	Y	Y	Y	Y	Y	Y	Y	Y	Y	Y	Y	N	Y	Y	Y	Y	Y	Y	Y	Y	Y	Y	Y
19	Details of currency of price adjustments for inflation or currency conversion are given.	N	N	N	N	N	N	N	Y	Y	N	Y	Y	Y	N	Y	Y	Y	N	N	Y	N	Y	Y	Y	NA
20	Details of any model used are given.	N	Y	Y	N	N	N	Y	Y	Y	Y	Y	N	N	N	Y	Y	Y	Y	Y	Y	Y	Y	Y	Y	Y
21	The choice of model used and the key parameters on which it is based are justified.	N	Y	Y	N	N	N	N	N	Y	Y	Y	N	N	N	Y	Y	Y	Y	Y	Y	Y	Y	Y	Y	Y
	Analysis and interpretation of results																									
22	Time horizon of costs and benefits is stated.	Y	Y	Y	Y	Y	Y	Y	Y	Y	Y	Y	Y	Y	Y	Y	Y	Y	Y	Y	Y	Y	Y	Y	Y	Y
23	The discount rate(s) is stated.	Y	Y	NA	Y	Y	N	Y	Y	N	Y	Y	Y	Y	N	N	Y	N	Y	Y	Y	Y	N	Y	Y	Y
24	The choice of discount rate(s) is justified.	Y	N	Y	N	N	N	N	Y	N	Y	Y	Y	Y	N	N	N	N	N	Y	Y	N	N	Y	Y	N
25	An explanation is given if costs and benefits are not discounted.	NA	NA	Y	NA	NA	N	NA	Y	N	NA	NA	NA	Y	N	N	NA	N	NA	Y	Y	NA	N	Y	NA	NA
26	Details of statistical tests and confidence intervals are given for stochastic data.	NA	Y	NA	NA	NA	NA	NA	NA	NA	NA	NA	NA	NA	N	NA	NA	NA	NA	NA	NA	NA	NA	NA	NA	NA
27	The approach to sensitivity analysis is given.	Y	Y	Y	Y	Y	Y	Y	Y	Y	Y	Y	Y	Y	N	Y	Y	Y	Y	Y	Y	Y	Y	Y	N	Y
28	The choice of variables for sensitivity analysis is justified.	N	Y	Y	Y	Y	N	N	N	N	N	N	Y	Y	N	N	Y	N	N	N	N	N	N	N	N	Y
29	The ranges over which the variables are varied are justified.	N	N	Y	Y	Y	N	Y	N	Y	N	N	Y	Y	N	N	Y	N	N	Y	N	N	N	Y	N	N
30	Relevant alternatives are compared.	Y	Y	Y	Y	Y	Y	Y	Y	Y	Y	Y	Y	Y	N	Y	Y	N	Y	Y	N	Y	N	N	Y	Y
31	Incremental analysis is reported.	Y	Y	Y	Y	Y	Y	Y	Y	Y	Y	Y		Y	N	Y	Y	Y	Y	Y	N	Y	Y	Y	N	Y

32	Major outcomes are presented in a disaggregated as well as aggregated form.	N	Y	Y	N	N	N	Y	Y	Y	Y	Y	Y	Y	N	Y	Y	Y	Y	Y	N	Y	Y	Y	NA	Y
33	The answer to the study question is given.	Y	Y	Y	Y	Y	Y	Y	Y	Y	Y	Y	Y	Y	Y	Y	Y	Y	Y	Y	Y	Y	Y	Y	Y	Y
34	Conclusions follow from the data reported.	Y	Y	Y	NA	NA	Y	Y	Y	Y	Y	Y	Y	Y	Y	Y	Y	Y	Y	Y	Y	Y	Y	Y	NA	Y
35	Conclusions are accompanied by the appropriate caveats.	Y	Y	Y	NA	NA	Y	Y	Y	Y	N	Y	Y	Y	N	Y	Y	Y	Y	Y	Y	Y	Y	Y	NA	Y
	Phillips et al. (2004) Checklist																									
	Statement of decision problem/objective																									
1	Is there a clear statement of the decision problem?	Y	Y	Y	Y	Y	Y	Y	Y	Y	Y	Y	Y	Y	Y	Y	Y	Y	Y	Y	Y	Y	Y	Y	Y	Y
2	Is the objective of the evaluation and model specified and consistent with the stated decision problem?	Y	Y	Y	Y	Y	Y	Y	Y	Y	Y	Y	Y	Y	Y	Y	Y	Y	Y	Y	Y	Y	Y	Y	Y	Y
3	Is the primary decision-maker specified?	N	N	Y	N	N	N	N	Y	N	N	N	Y	N	N	N	Y	N	Y	N	N	N	Y	N	Y	N
	Statement of scope/perspective																									
4	Is the perspective of the model stated clearly?	Y	Y	Y	Y	Y	Y	Y	Y	N	Y	Y	Y	Y	Y	Y	Y	Y	Y	Y	Y	Y	Y	Y	Y	Y
5	Are the model inputs consistent with the stated perspective?	N	Y	Y	Y	Y	Y	Y	Y	NA	Y	Y	Y	Y	Y	Y	Y	Y	Y	N	Y	Y	Y	Y	Y	Y
6	Has the scope of the model been stated and justified?	Y	Y	Y	Y	Y	Y	Y	Y	Y	Y	Y	Y	Y	Y	Y	Y	Y	Y	Y	Y	Y	Y	Y	Y	Y
7	Are the outcomes of the model consistent with the perspective, scope and overall objective of the model?	N	Y	Y	Y	Y	Y	Y	Y	NA	Y	Y	Y	Y	Y	Y	Y	Y	Y	Y	Y	Y	Y	Y	Y	Y
	Rationale for structure																									
8	Is the structure of the model consistent with a coherent theory of the health condition under	N	Y	Y	Y	Y	Y	Y	N	Y	Y	Y	Y	N	N	Y	Y	Y	Y	Y	Y	Y	Y	N	Y	Y

	evaluation?																									
9	Are the sources of data used to develop the structure of the model specified?	N	Y	Y	N	N	N	Y	Y	N	N	Y	N	N	N	N	Y	N	N	N	Y	Y	N	N	Y	Y
10	Are the causal relationships described by the model structure justified appropriately?	N	N	Y	N	N	N	N	N	N	Y	Y	NA	N	N	Y	Y	Y	Y	Y	Y	N	Y	N	NA	N
	Structural assumptions																									
11	Are the structural assumptions transparent and justified?	N	Y	Y	N	N	N	N	N	N	N	Y	Y	Y	N	Y	Y	Y	Y	Y	N	N	N	N	Y	Y
12	Are the structural assumptions reasonable given the overall objective, perspective and scope of the model?	NA	Y	Y	N	N	Y	N	N	N	Y	Y	Y	N	NA	Y	Y	Y	Y	Y	N	N	N	N	N	Y
	Strategies/comparators																									
13	Is there a clear definition of the options under evaluation?	Y	Y	Y	Y	Y	Y	Y	Y	Y	Y	Y	Y	Y	Y	Y	Y	Y	Y	Y	Y	Y	Y	Y	Y	Y
14	Have all feasible and practical options been evaluated?	Y	Y	N	N	N	N	N	N	N	N	Y	N	N	N	N	Y	N	Y	N	N	N	N	N	Y	Y
15	Is there justification for the exclusion of feasible options?	NA	NA	N	N	N	N	N	N	N	N	Y	N	N	N	N	NA	Y	NA	N	N	N	N	N	NA	NA
	Model type																									
16	Is the chosen model type appropriate given the decision problem and specified causal relationships within the model?	N	Y	Y	N	N	N	Y	N	Y	Y	Y	Not clear	N	N	N	Y	N	Y	N	N	Y	N	Y	Y	Y
	Time horizon																									
17	Is the time horizon of the model sufficient to reflect all important differences between options?	Y	N	N	Y	N	N	Y	N	N	N	Y	Y	N	N	N	N	N	N	N	N	N	N	N	Y	Y
18	Are the time horizon of the model, the duration of	Y	Y	Y	N	N	N	N	Y	Y	N	Y	Y	Y	N	N	Y	N	N	N	N	Y	Y	Y	Y	N

	treatment and the duration of treatment effect described and justified?																									
	Disease states/pathways																									
19	Do the disease states (state transition model) or the pathways (decision tree model) reflect the underlying biological process of the disease in question and the impact of interventions?	Y	Y	Y	Y	N	N	Y	Y	Y	Y	Y	Y	Y	N	Y	Y	Y	Y	Y	Y	Y	N	Y	Y	Y
	Cycle length																									
20	Is the cycle length defined and justified in terms of the natural history of disease?	N	Y	Y	N	N	N	Y	N	N	Y	Y	NA	NA	NA	N	Y	N	Y	N	N	Y	N	Y	Y	Y
	Data identification																									
21	Are the data identification methods transparent and appropriate given the objectives of the model?	N	N	Y	N	N	N	N	Y	N	N	Y	Y	Y	N	Y	Y	N	Y	Y	Y	N	N	N	N	N
22	Where choices have been made between data sources, are these justified appropriately?	N	N	N	N	NA	N	N	N	N	N	Y	NA	N	N	N	Y	N	N	N	N	NA	N	N	NA	N
23	Has particular attention been paid to identifying data for the important parameters in the model?	N	Y	Y	N	Y	N	Y	Y	Y	Y	Y	Y	Y	Y	Y	Y	N	Y	Y	Y	Y	Y	Y	Y	Y
24	Has the quality of the data been assessed appropriately?	N	N	Y	N	N	N	N	N	N	N	N	N	N	N	N	Y	N	Y	N	N	N	N	N	N	N
25	Where expert opinion has been used, are the methods described and justified?	Y	N	Y	N	NA	NA	N	N	NA	NA	NA	NA	NA	NA	NA	Y	NA	N	N	NA	NA	N	NA	NA	Y
	Data modelling																									
26	Is the data modelling methodology based on	N	Y	Y	Y	N	Y	Y	Y	Y	Y	Y	N	N	N	Y	Y	Y	Y	Y	Y	Y	Y	Y	Y	Y

	justifiable statistical and epidemiological techniques?																									
	Baseline data																									
27	Is the choice of baseline data described and justified?	Y	Y	Y	N	N	N	N	Y	Y	Y	Y	Y	Y	Y	Y	Y	Y	Y	Y	Y	Y	Y	Y	Y	Y
28	Are transition probabilities calculated appropriately?	N	Y	Y	N	N	N	Y	Y	Y	Y	Y	N	Y	Y	Y	Y	Y	Y	Y	Y	Y	Y	Y	N	Y
29	Has a half-cycle correction been applied to both cost and outcome? If not, has this omission been justified?	N	N	N	N	N	N	N	N	N	N	N	N	N	N	N	N	N	N	N	N	N	N	Y	N	Y
	Treatment effects																									
30	If relative treatment effects have been derived from trial data, have they been synthesised using appropriate techniques?	NA	Y	Y	N	N	N	NA	N	N	N	Y	Y	N	N	Y	Y	Y	Y	Y	Y	NA	N	N	Y	N
31	Have the methods and assumptions used to extrapolate shortterm results to final outcomes been documented and justified?	NA	N	N	N	NA	N	N	NA	N	N	Y	Y	N	Y	N	N	N	N	N	N	N	N	N	N	N
32	Have alternative assumptions been explored through sensitivity analysis?	Y	N	N	Y	N	Y	N	N	Y	N	Y	Y	N	N	N	N	N	N	N	N	N	N	N	N	N
33	Have assumptions regarding the continuing effect of treatment once treatment is complete been documented and justified? Have alternative assumptions been explored through sensitivity analysis?	N	N	Y	N	N	N	N	N	N	N	Y	N	N	N	N	N	N	N	N	N	N	N	N	N	N
	Costs																									
34	Are the costs incorporated into the	N	Y	Y	N	N	N	N	Y	Y	Y	Y	Y	Y	Y	Y	Y	Y	Y	Y	Y	Y	Y	Y	Y	N

	model justified?																									
35	Has the source for all costs been described?	Y	Y	Y	Y	N	N	N	Y	Y	Y	Y	Y	Y	Y	Y	Y	Y	Y	Y	Y	Y	Y	Y	Y	Y
36	Have discount rates been described and justified given the target decision-maker?	Y	Y	NA	Y	Y	N	Y	Y	N	Y	Y	Y	Y	N	Y	Y	N	Y	Y	Y	Y	N	Y	Y	Y
	Quality of life weights (utilities)																									
37	Are the utilities incorporated into the model appropriate?	N	N	Y	Y	N	Y	Y	Y	Y	Y	Y	Y	Y	NA	Y	Y	Y	Y	Y	N	Y	Y	Y	Y	Y
38	Is the source for the utility weights referenced?	Y	Y	Y	Y	Y	Y	Y	Y	Y	Y	Y	Y	Y	NA	Y	Y	Y	Y	Y	Y	Y	Y	Y	Y	Y
39	Are the methods of derivation for the utility weights justified?	N	Y	N	N	N	N	N	Y	Y	Y	Y	Y	N	NA	N	Y	N	Y	Y	N	Y	Y	N	Y	N
	Data incorporation																									
40	Have all data incorporated into the model been described and referenced in sufficient detail?	Y	N	Y	N	N	N	N	Y	N	Y	Y	Y	N	N	Y	Y	Y	Y	Y	Y	Y	Y	Y	N	Y
41	Has the use of mutually inconsistent data been justified (i.e. are assumptions and choices appropriate)?	N	Y	N	N	N	N	N	N	Y	N	Y	N	N	N	Y	Y	N	Y	Y	N	N	Y	N	N	Y
42	Is the process of data incorporation transparent?	N	Y	Y	N	N	N	N	N	N	N	Y	Y	N	N	Y	Y	Y	Y	Y	Y	N	Y	Y	NA	Y
43	If data have been incorporated as distributions, has the choice of distribution for each parameter been described and justified?	NA	NA	Y	NA	NA	NA	NA	NA	NA	NA	N	Y	Y	NA	NA	NA	NA	NA	NA	Y	Y	N	NA	NA	NA
44	If data have been incorporated as distributions, is it clear that second order uncertainty is reflected?	NA	NA	N	NA	NA	NA	NA	NA	NA	NA	N	N	N	NA	NA	NA	NA	NA	NA	Y	Y	N	NA	NA	NA
	Assessment of																									

	uncertainty																									
45	Have the four principal types of uncertainty been addressed?	N	N	N	N	N	N	N	N	N	N	N	N	N	N	N	N	N	Y	N	N	N	N	N	N	N
46	If not, has the omission of particular forms of uncertainty been justified?	N	N	N	N	N	N	N	N	N	N	N	N	N	N	N	N	N	NA	N	N	N	N	N	N	N
	Methodological																									
47	Have methodological uncertainties been addressed by running alternative versions of the model with different methodological assumptions?	N	N	N	N	N	N	N	N	N	N	Y	Y	N	N	N	Y	N	Y	Y	N	N	N	N	NA	N
	Structural																									
48	Is there evidence that structural uncertainties have been addressed via sensitivity analysis?	N	N	N	Y	N	Y	Y	Y	Y	N	N	Y	Y	N	Y	Y	N	Y	Y	Y	Y	Y	N	NA	N
	Heterogeneity		N																							
49	Has heterogeneity been dealt with by running the model separately for different subgroups?	N		N	N	N	N	N	N	N	Y	N	N	N	N	N	N	N	N	N	N	N	N	N	N	N
	Parameter																									
50	Are the methods of assessment of parameter uncertainty appropriate?	N	Y	Y	N	N	N	N	N	Y	Y	Y	Y	Y	NA	N	Y	N	Y	Y	Y	Y	Y	Y	NA	Y
51	If data are incorporated as point estimates, are the ranges used for sensitivity analysis stated clearly and justified?	N	Y	Y	N	N	N	N	N	N	N	N	N	Y	NA	N	Y	N	N	Y	N	N	N	Y	NA	Y
	Internal consistency																									
52	Is there evidence that the mathematical logic of the model has	N	N	N	N	N	N	N	N	N	N	Y	N	N	N	N	N	N	Y	N	Y	N	N	N	Y	N

	been tested thoroughly before use?																									
	External consistency																									
53	Are any counterintuitive results from the model explained and justified?	N	NA	Y	N	N	N	N	Y	NA	NA	Y	N	Y	N	Y	Y	NA	Y	Y	Y	NA	Y	NA	NA	NA
54	If the model has been calibrated against independent data, have any differences been explained and justified?	NA	NA	NA	NA	NA	NA	NA	NA	NA	NA	NA	NA	NA	NA	NA	NA	NA	NA	NA	NA	NA	NA	NA	NA	NA
55	Have the results of the model been compared with those of previous models and any differences in results explained?	N	N	Y	N	N	N	N	Y	Y	Y	Y	Y	Y	N	Y	Y	Y	Y	Y	Y	Y	Y	Y	NA	N

Table 2 Risk of bias assessment for studies on Ulcerative Colitis

	Reference	Panes et al. (2007)	Buckland & Bodger (2008)	Yen et al. (2008)	Tsai et al. (2008)	Connolly et al. (a) (2009)	Connolly et al. (b) (2009)	Xie et al. (2009)	Brereton et al. (2010)	Punekar et al. (2010)	Prenzler et al. (2011)	Connolly et al. (2012)	Park et al. (2012)	Saini et al. (2012)	Chaudhary & Fan (2013)	Connolly et al. (2014)	Essat (2014)	Archer et al. (NICE) - MSD model for golimumab and infliximab (2014)	Archer et al. (NICE) - AbbVie model for adalimumab (2014)	Beilman Et al. (2016)	Stawowczyk et al. (2016) golimumab&standard care vs. Standard care alone	Stawowczyk et al. (2016) adalmimumab&standard care vs. Standard care alone	Stawowczyk et al. (2016) - infliximab&standard care vs. Standard care alone	Yokomizo et al. (2016)	Tappenden et al. (2016)	Wilson (2017)
	Drummond et al. (1996) checklist																									
	Study design																									
1	The research question is stated.	Y	Y	Y	Y	Y	Y	Y	Y	Y	Y	Y	Y	Y	Y	Y	Y	Y	Y	Y	Y	Y	Y	Y	Y	Y
2	The economic importance of the research question is stated.	Y	Y	Y	Y	Y	Y	Y	Y	Y	Y	Y	Y	Y	Y	Y	Y	Y	Y	Y	Y	Y	Y	Y	Y	Y
3	The viewpoint(s) of the analysis are clearly stated and justified.	Y	Y	Y	Y	Y	Y	Y	Y	Y	Y	Y	Y	Y	Y	Y	Y	Y	Y	Y	Y	Y	Y	Y	Y	Y
4	The rationale for choosing alternative programmes or interventions compared is stated.	Y	Y	Y	Y	Y	Y	Y	Y	Y	Y	Y	Y	Y	Y	Y	Y	Y	Y	Y	Y	Y	Y	Y	Y	Y
5	The alternatives being compared are clearly described.	Y	Y	Y	Y	Y	Y	Y	Y	Y	Y	Y	Y	Y	Y	Y	Y	Y	Y	Y	Y	Y	Y	Y	Y	Y

6	The form of economic evaluation used is stated.	Y	Y	Y	Y	Y	Y	Y	Y	Y	Y	Y	Y	Y	Y	Y	Y	Y	Y	Y	Y	Y	Y	Y	Y	Y
7	The choice of form of economic evaluation is justified in relation to the questions addressed.	Y	Y	Y	Y	Y	Y	Y	Y	Y	Y	Y	Y	Y	Y	Y	N	Y	Y	Y	Y	Y	Y	Y	Y	Y
	Data collection																									
8	The source(s) of effectiveness estimates used are stated.	Y	Y	Y	Y	Y	Y	Y	Y	Y	Y	Y	Y	Y	Y	Y	Y	Y	Y	N	Y	Y	Y	Y	Y	Y
9	Details of the design and results of effectiveness study are given (if based on a single study).	NA	NA	N	NA	Y	N	NA	NA	NA	NA	N	NA	NA	NA	Y	Y	NA	NA	NA	NA	Y	Y	Y	NA	NA
10	Details of the methods of synthesis or meta-analysis of estimates are given (if based on a synthesis of a number of effectiveness studies).	N	Y	N	N	NA	NA	Y	N	Y	N	NA	Y	N	N	NA	NA	Y	Y	N	N	NA	NA	NA	Y	Y
11	The primary outcome measure(s) for the economic evaluation are clearly stated.	Y	Y	Y	Y	Y	Y	Y	N	Y	Y	Y	Y	Y	Y	Y	Y	Y	Y	Y	Y	Y	Y	Y	Y	Y
12	Methods to value benefits are stated.	Y	Y	Y	Y	Y	Y	Y	N	Y	Y	Y	Y	Y	Y	N	Y	Y	Y	N	Y	Y	Y	Y	Y	N
13	Details of the subjects from whom valuations were obtained were given.	N	N	N	N	Y	N	N	N	N	Y	N	N	N	N	N	N	N	N	N	N	N	N	N	N	N
14	Productivity changes (if included) are reported separately.	NA	NA	NA	NA	NA	NA	NA	NA	NA	NA	NA	NA	NA	NA	NA	NA	NA	NA	NA	Y	Y	NA	NA	NA	NA
15	The relevance of productivity changes to the study question is discussed.	N	N	N	Y	Y	N	N	N	Y	N	Y	N	N	Y	N	N	N	N	Y	Y	Y	N	N	N	N
16	Quantities of resource use are reported separately from their unit costs.	Y	N	N	Y	N	N	N	N	Y	N	N	N	N	Y	N	N	N	N	N	Y	N	N	N	Y	Y
17	Methods for the estimation of quantities and unit costs are described.	Y	Y	Y	Y	Y	Y	Y	Y	Y	Y	Y	Y	Y	Y	Y	Y	Y	Y	Y	Y	Y	Y	Y	Y	Y
18	Currency and price data are recorded.	Y	Y	Y	Y	Y	Y	Y	Y	Y	Y	Y	Y	Y	Y	Y	Y	Y	Y	Y	Y	Y	Y	Y	Y	Y
19	Details of currency of price adjustments for inflation or currency conversion are given.	N	N	N	N	N	N	N	N	N	N	N	N	Y	Y	Y	N	N	N	N	Y	Y	Y	N	Y	Y

20	Details of any model used are given.	Y	Y	Y	Y	Y	Y	Y	Y	Y	Y	Y	Y	Y	Y	Y	Y	Y	Y	Y	Y	Y	Y	Y	Y	Y
21	The choice of model used and the key parameters on which it is based are justified.	N	Y	Y	Y	N	N	Y	Y	Y	Y	Y	Y	Y	Y	Y	N	Y	Y	Y	Y	Y	Y	Y	Y	Y
	Analysis and interpretation of results																									
22	Time horizon of costs and benefits is stated.	Y	Y	Y	Y	Y	N	Y	Y	Y	Y	Y	Y	Y	Y	Y	Y	Y	Y	Y	Y	Y	Y	Y	Y	Y
23	The discount rate(s) is stated.	Y	N	Y	Y	N	N	Y	Y	Y	Y	Y	Y	Y	Y	Y	Y	Y	Y	Y	Y	Y	Y	N	Y	Y
24	The choice of discount rate(s) is justified.	Y	N	N	Y	N	N	N	Y	Y	Y	Y	Y	Y	N	Y	Y	Y	Y	Y	N	N	Y	N	Y	Y
25	An explanation is given if costs and benefits are not discounted.	Y	N	Y	NA	N	N	NA	NA	NA	NA	NA	NA	NA	NA	Y	NA	NA	NA	NA	NA	NA	NA	N	NA	NA
26	Details of statistical tests and confidence intervals are given for stochastic data.	NA	NA	NA	NA	NA	NA	NA	NA	NA	NA	NA	NA	NA	NA	NA	NA	NA	NA	NA	NA	NA	NA	NA	NA	NA
27	The approach to sensitivity analysis is given.	Y	Y	Y	Y	Y	Y	Y	Y	Y	Y	Y	Y	Y	Y	Y	Y	Y	Y	Y	Y	Y	Y	Y	Y	Y
28	The choice of variables for sensitivity analysis is justified.	N	Y	Y	N	Y	N	Y	Y	Y	N	N	Y	Y	N	N	N	Y	N	N	N	N	N	N	Y	N
29	The ranges over which the variables are varied are justified.	N	Y	Y	N	Y	N	Y	Y	Y	N	N	Y	N	N	N	N	N	N	Y	N	N	Y	N	Y	Y
30	Relevant alternatives are compared.	N	N	Y	Y	Y	N	Y	Y	Y	Y	Y	Y	N	Y	Y	Y	N	N	N	N	N	N	N	Y	Y
31	Incremental analysis is reported.	Y	Y	Y	Y	Y	Y	Y	Y	Y	Y	Y	Y	Y	Y	Y	Y	N	Y	Y	Y	Y	Y	Y	Y	Y
32	Major outcomes are presented in a disaggregated as well as aggregated form.	Y	N	Y	Y	Y	N	Y	Y	Y	Y	Y	Y	Y	Y	Y	N	Y	Y	Y	Y	Y	Y	Y	Y	Y
33	The answer to the study question is given.	Y	Y	Y	Y	Y	Y	Y	Y	Y	Y	Y	Y	Y	Y	Y	Y	Y	Y	Y	Y	Y	Y	Y	Y	Y
34	Conclusions follow from the data reported.	Y	Y	Y	Y	Y	Y	Y	Y	Y	Y	Y	Y	Y	Y	Y	NA	NA	NA	Y	Y	Y	Y	Y	Y	Y
35	Conclusions are accompanied by the appropriate caveats.	Y	Y	Y	N	Y	Y	Y	Y	Y	Y	Y	Y	Y	Y	Y	NA	NA	NA	Y	Y	Y	Y	Y	Y	Y
	Phillips et al. (2004) Checklist																									
	Statement of decision problem/objective																									
1	Is there a clear statement of the decision problem?	Y	Y	Y	Y	Y	Y	Y	Y	Y	Y	Y	Y	Y	Y	Y	Y	Y	Y	Y	Y	Y	Y	Y	Y	Y

2	Is the objective of the evaluation and model specified and consistent with the stated decision problem?	Y	Y	Y	Y	Y	Y	Y	Y	Y	Y	Y	Y	Y	Y	Y	Y	Y	Y	Y	Y	Y	Y	Y	Y	Y
3	Is the primary decision-maker specified?	N	Y	N	Y	Y	Y	Y	Y	N	Y	N	N	Y	Y	N	Y	Y	Y	N	N	N	N	N	Y	Y
	Statement of scope/perspective																									
4	Is the perspective of the model stated clearly?	Y	Y	Y	Y	Y	Y	Y	Y	Y	Y	Y	Y	Y	Y	Y	Y	Y	Y	Y	Y	Y	Y	Y	Y	Y
5	Are the model inputs consistent with the stated perspective?	Y	Y	Y	Y	Y	Y	Y	Y	Y	Y	Y	N	Y	Y	Y	Y	Y	Y	Y	Y	Y	Y	Y	Y	Y
6	Has the scope of the model been stated and justified?	Y	Y	Y	Y	Y	Y	Y	Y	Y	Y	Y	Y	Y	Y	Y	Y	Y	Y	Y	Y	Y	Y	Y	Y	Y
7	Are the outcomes of the model consistent with the perspective, scope and overall objective of the model?	Y	Y	Y	Y	Y	Y	Y	Y	Y	Y	Y	N	Y	Y	Y	Y	Y	Y	N	Y	Y	Y	Y	Y	Y
	Rationale for structure																									
8	Is the structure of the model consistent with a coherent theory of the health condition under evaluation?	N	Y	Y	N	Y	Y	Y	Y	Y	Y	Y	Y	Y	Y	Y	Y	Y	N	Y	Y	Y	Y	Y	Y	Y
9	Are the sources of data used to develop the structure of the model specified?	N	N	Y	N	Y	N	N	N	Y	Y	Y	Y	N	Y	Y	N	N	N	Y	N	N	N	N	Y	Y
10	Are the causal relationships described by the model structure justified appropriately?	N	N	N	N	Y	N	Y	Y	N	Y	N	N	Y	N	N	NA	N	N	N	Y	Y	Y	N	Y	Y
	Structural assumptions																									
11	Are the structural assumptions transparent and justified?	N	N	Y	N	N	N	Y	N	N	N	N	N	Y	Y	N	Y	N	N	N	Y	Y	Y	N	Y	N
12	Are the structural assumptions reasonable given the overall objective, perspective and scope of the model?	N	N	Y	N	N	N	Y	NA	N	NA	NA	N	Y	Y	NA	Y	N	N	N	N	N	Y	N	Y	Y
	Strategies/comparators																									
13	Is there a clear definition of the options under evaluation?	Y	Y	Y	Y	Y	Y	Y	Y	Y	Y	Y	Y	Y	Y	Y	Y	Y	Y	Y	Y	Y	Y	Y	Y	Y
14	Have all feasible and practical options been evaluated?	N	N	Y	N	Y	Y	Y	N	Y	N	Y	Y	N	Y	N	Y	N	N	N	N	N	N	N	Y	Y

15	Is there justification for the exclusion of feasible options?	N	N	NA	N	Y	Y	NA	Y	NA	Y	NA	NA	Y	NA	Y	NA	N	N	N	N	N	N	N	Y	NA
	Model type																									
16	Is the chosen model type appropriate given the decision problem and specified causal relationships within the model?	Y	Y	Y	Y	Y	Y	Y	Y	Y	Y	Y	Y	Y	N	Y	N	Y	Y	Y	Y	Y	Y	N	Y	Y
	Time horizon																									
17	Is the time horizon of the model sufficient to reflect all important differences between options?	N	N	N	N	N	N	N	N	N	N	N	Y	N	N	N	N	N	N	N	Y	Y	Y	N	Y	Y
18	Are the time horizon of the model, the duration of treatment and the duration of treatment effect described and justified?	Y	Y	Y	Y	Y	N	Y	Y	Y	N	N	Y	N	N	N	N	N	N	N	Y	Y	Y	N	Y	Y
	Disease states/pathways																									
19	Do the disease states (state transition model) or the pathways (decision tree model) reflect the underlying biological process of the disease in question and the impact of interventions?	Y	Y	Y	Y	Y	Y	Y	Y	Y	Y	Y	N	Y	Y	Y	Y	Y	Y	Y	Y	Y	Y	Y	Y	Y
	Cycle length																									
20	Is the cycle length defined and justified in terms of the natural history of disease?	NA	NA	Y	Y	NA	Y	Y	Y	Y	Y	Y	Y	Y	Y	Y	Y	Y	Y	Y	Y	Y	Y	NA	Y	Y
	Data identification																									
21	Are the data identification methods transparent and appropriate given the objectives of the model?	N	N	Y	N	Y	Y	Y	N	N	Y	Y	Y	N	N	Y	Y	Y	N	N	N	Y	N	Y	Y	Y
22	Where choices have been made between data sources, are these justified appropriately?	N	N	Y	N	NA	NA	N	NA	N	Y	NA	N	N	N	NA	NA	N	NA	NA	NA	NA	N	NA	NA	NA
23	Has particular attention been paid to identifying data for the	Y	Y	Y	Y	Y	NA	Y	Y	Y	Y	Y	Y	Y	N	NA	Y	Y	NA	N	Y	Y	Y	Y	Y	Y

	important parameters in the model?																									
24	Has the quality of the data been assessed appropriately?	N	N	N	N	N	N	N	N	N	N	N	N	N	N	N	N	N	N	N	N	N	N	N	Y	N
25	Where expert opinion has been used, are the methods described and justified?	N	Y	N	NA	NA	NA	NA	NA	N	NA	NA	N	NA	NA	NA	NA	N	NA	Y	N	N	N	NA	N	N
	Data modelling																									
26	Is the data modelling methodology based on justifiable statistical and epidemiological techniques?	N	Y	Y	Y	Y	N	Y	Y	Y	Y	Y	Y	Y	Y	Y	Y	Y	Y	Y	Y	Y	Y	Y	Y	Y
	Baseline data																									
27	Is the choice of baseline data described and justified?	Y	Y	Y	Y	Y	Y	Y	Y	Y	Y	Y	Y	Y	Y	Y	Y	Y	Y	Y	Y	Y	Y	Y	Y	Y
28	Are transition probabilities calculated appropriately?	Y	Y	Y	Y	Y	Y	Y	Y	Y	Y	Y	Y	Y	Y	Y	N	N	N	Y	Y	Y	Y	Y	Y	Y
29	Has a half-cycle correction been applied to both cost and outcome? If not, has this omission been justified?	N	N	N	N	N	N	N	N	N	N	N	N	N	N	N	N	N	N	N	N	N	N	N	N	N
	Treatment effects																									
30	If relative treatment effects have been derived from trial data, have they been synthesised using appropriate techniques?	Y	Y	Y	N	N	Y	Y	Y	Y	Y	Y	Y	NA	Y	Y	Y	Y	Y	N	Y	Y	N	Y	Y	Y
31	Have the methods and assumptions used to extrapolate shortterm results to final outcomes been documented and justified?	NA	NA	Y	Y	NA	NA	N	N	N	N	NA	N	N	NA	N	N	N	N	N	N	NA	N	Y	N	N
32	Have alternative assumptions been explored through sensitivity analysis?	Y	Y	Y	Y	Y	N	N	N	N	Y	N	N	N	N	N	N	N	N	N	N	N	N	N	Y	Y
33	Have assumptions regarding the continuing effect of treatment once treatment is complete been documented and justified? Have alternative assumptions been explored through sensitivity analysis?	NA	N	Y	N	N	N	N	N	Y	N	N	N	N	N	N	N	N	N	N	N	N	N	Y	N	Y

	Costs																									
34	Are the costs incorporated into the model justified?	Y	Y	Y	Y	Y	Y	Y	Y	Y	Y	Y	Y	Y	Y	Y	Y	Y	Y	Y	Y	Y	Y	Y	Y	Y
35	Has the source for all costs been described?	Y	Y	Y	Y	Y	Y	Y	Y	Y	Y	Y	Y	Y	Y	Y	Y	Y	Y	Y	Y	Y	Y	Y	Y	Y
36	Have discount rates been described and justified given the target decision-maker?	Y	N	Y	Y	Y	N	Y	Y	Y	Y	Y	Y	Y	Y	Y	Y	Y	Y	Y	Y	Y	Y	N	Y	Y
	Quality of life weights (utilities)																									
37	Are the utilities incorporated into the model appropriate?	NA	Y	Y	Y	Y	Y	Y	Y	Y	Y	Y	Y	Y	Y	Y	N	N	Y	Y	Y	Y	Y	NA	Y	N
38	Is the source for the utility weights referenced?	NA	Y	Y	Y	Y	Y	Y	Y	Y	Y	Y	Y	Y	Y	Y	Y	Y	Y	N	Y	Y	Y	NA	Y	Y
39	Are the methods of derivation for the utility weights justified?	NA	Y	Y	Y	Y	Y	N	N	Y	N	N	N	N	Y	N	N	N	N	N	Y	Y	Y	NA	Y	N
	Data incorporation																									
40	Have all data incorporated into the model been described and referenced in sufficient detail?	N	Y	Y	Y	Y	Y	Y	Y	Y	Y	N	N	Y	Y	N	Y	N	Y	N	Y	Y	Y	Y	Y	Y
41	Has the use of mutually inconsistent data been justified (i.e. are assumptions and choices appropriate)?	N	N	Y	N	N	N	Y	Y	N	N	N	N	N	N	N	Y	N	N	N	N	N	N	Y	Y	Y
42	Is the process of data incorporation transparent?	N	N	Y	N	N	N	Y	Y	Y	N	N	N	N	Y	N	NA	N	N	N	Y	Y	Y	Y	Y	Y
43	If data have been incorporated as distributions, has the choice of distribution for each parameter been described and justified?	NA	NA	NA	NA	N	NA	Y	NA	N	NA	NA	N	NA	NA	N	NA	NA	NA	NA	NA	NA	NA	NA	N	NA
44	If data have been incorporated as distributions, is it clear that second order uncertainty is reflected?	NA	NA	NA	NA	Y	NA	Y	NA	N	NA	NA	N	NA	NA	N	NA	NA	NA	NA	NA	NA	NA	NA	N	NA
	Assessment of uncertainty																									
45	Have the four principal types of uncertainty been addressed?	N	N	N	N	N	N	N	N	N	N	N	N	N	N	N	N	N	N	N	N	N	N	N	N	N
46	If not, has the omission of particular forms of uncertainty been justified?	N	N	N	N	N	N	N	N	N	N	N	N	N	N	N	N	N	N	N	N	N	N	N	N	N
	Methodological																									

47	Have methodological uncertainties been addressed by running alternative versions of the model with different methodological assumptions?	N	N	Y	Y	Y	Y	Y	N	N	N	Y	N	N	Y	N	Y	N	N	N	N	N	N	N	N	N
	Structural																									
48	Is there evidence that structural uncertainties have been addressed via sensitivity analysis?	N	N	N	Y	N	N	Y	Y	Y	N	N	N	N	N	N	Y	N	N	Y	Y	Y	Y	N	Y	Y
	Heterogeneity																									
49	Has heterogeneity been dealt with by running the model separately for different subgroups?	N	N	N	Y	N	N	N	N	N	N	Y	N	N	N	N	Y	N	N	N	N	N	N	N	Y	N
	Parameter																									
50	Are the methods of assessment of parameter uncertainty appropriate?	N	Y	Y	Y	Y	Y	Y	Y	Y	Y	Y	Y	Y	Y	Y	Y	Y	Y	Y	Y	Y	Y	Y	Y	Y
51	If data are incorporated as point estimates, are the ranges used for sensitivity analysis stated clearly and justified?	Y	Y	Y	N	Y	Y	Y	Y	N	N	N	Y	Y	N	Y	NA	N	N	Y	Y	Y	Y	Y	Y	Y
	Internal consistency																									
52	Is there evidence that the mathematical logic of the model has been tested thoroughly before use?	N	N	N	N	N	N	N	N	N	Y	Y	N	N	Y	Y	N	N	N	Y	Y	Y	Y	N	Y	Y
	External consistency																									
53	Are any counterintuitive results from the model explained and justified?	NA	NA	NA	NA	NA	NA	Y	Y	NA	NA	NA	NA	Y	NA	Y	NA	NA	NA	N	NA	NA	NA	Y	NA	NA
54	If the model has been calibrated against independent data, have any differences been explained and justified?	NA	NA	NA	NA	NA	NA	NA	NA	NA	NA	NA	NA	NA	NA	NA	NA	NA	NA	N	NA	NA	NA	NA	NA	NA
55	Have the results of the model	Y	N	N	Y	N	N	Y	Y	N	N	Y	Y	Y	Y	N	NA	NA	NA	N	Y	Y	Y	N	Y	Y

	been compared with those of previous models and any differences in results explained?																									
